# Report of the 1st International Electronic Conference on Toxins (IECT2021), 16–31 January 2021

**DOI:** 10.3390/toxins13040273

**Published:** 2021-04-09

**Authors:** Jay W. Fox

**Affiliations:** Department of Microbiology, University of Virginia, Charlottesville, VA 22904, USA; jwf8x@virginia.edu

## 1. Preface

The 1st International Electronic Conference on Toxins (IECT2021) was successfully held online by https://sciforum.net/conference/IECT2021 from 16 to 31 January 2021. The mission of this conference is to provide a platform for scientists working on toxins from all organisms to present the latest concepts under research on these toxins and for all to compare and contrast the actions of toxins. The potential uses of toxins for the benefit of science, as well as for humankind, are key concepts up for discussion.

IECT2021 is led by *Toxins* Editor-in-Chief Prof. Dr. Jay Fox (University of Virginia, USA) and includes six section chairs from the editorial board of *Toxins*: Dr. Bryan Grieg Fry (University of Queensland, Australia; in charge of the contributions related to animal venoms), Prof. Dr. Sarah De De Saege (Ghent University, Belgium; in charge of the contributions related to mycotoxins), Prof. Dr. Michel R. Popoff (Institut Pasteur, France; in charge of the contributions related to bacterial toxins), Prof. Dr. Joachim Jankowski (University Hospital RWTH, Germany; in charge of the contributions related to uremic toxins), Prof. Dr. Mary Fletcher (University of Queensland, Australia; in charge of the contributions related to plant toxins), and Dr. Panagiota Katikou (Ministry of Rural Development and Food, Greece; in charge of the contributions related to marine and freshwater toxins).

More than 400 researchers participated in the conference in the form of paper/poster submissions. Four online sessions were successfully held during the conference, where 17 speakers gave speeches on their latest research and around 300 researchers watched the sessions online via Zoom or YouTube. Five prizes were awarded:

Best Paper Award 1: Suntravat, M., et al. “Profiling of Signaling Pathways in Human Dermal Blood and Lymphatic Endothelial Cells Induced by Snake Venom Cysteine-Rich Secretory Protein (svCRiSP) from North American snakes” (Section 5.7)

Best Paper Award 2: Dąbrowski, M., et al. “The Enteric Nerve System as Target of Regulated and Emerging Food-Associated Mycotoxins” (Section 5.2)

Best Poster Award 1: Avraamides, C., et al. “Linear Scorpion Peptides: An Unex-plored Pool for Peptide Hydrogels” (Section 6.9)

Best Poster Award 2: Wirén, C., et al. “Cyclodextrins as Capture Agents of Lipophilic Marine Toxins” (Section 7.9)

Best Oral Presentation Award: Vaiyapuri, S. (Chair); Gutiérrez, Z.M., and Laustsen A.H. “Unique Approaches to Tackle Snakebite-Induced Deaths and Disabilities”

## 2. Scientific Committee

Dr. Hang Fai (Henry) KWOK (University of Macau, China)

Dr. Sakthivel Vaiyapuri (University of Reading, UK)

Dr. Mettine H.A. Bos (Leiden University Medical Center, the Netherlands)

Dr. Irina Vetter (University of Queensland, Australia)

Dr. Ronald A. Jenner (Natural History Museum, UK)

Prof. Dr. Holger Scheib (University of Queensland, Australia)

Dr. Ana Maria Moura Da Silva (Instituto Butantan, Brazil)

Prof. Dr. Alberto Ritieni (University of Naples Federico II, Italy)

Dr. Daniela Eliza Marin (National Institut for Research and Development for Biology and Animal Nutrition, Romania)

Dr. Antonio Moretti (National Research Council (CNR), Italy)

Dr. Ting Zhou (Agriculture and Agri-Food Canada, Canada)

Prof. Dr. Laura Gámiz Gracia (University of Granada, Spain)

Dr. Ludger Johannes (PSL Research University, France)

Dr. Angela Melton-Celsa (Uniformed Services University, USA)

Prof. Dr. Keith E. Weaver (University of South Dakota, USA)

Prof. Dr. Tiina Rekand (Haukeland University Hospital, Norway)

Dr. Xiaohua He (USDA, USA)

Prof. Dr. Peter Šebo (Academy of Sciences of the Czech Republic, Czech Republic)

Dr. Yehu Moran (Hebrew University of Jerusalem, Israel)

Prof. Dr. Ángeles Jos (University of Sevilla, Spain)

Prof. Dr. Tibor Fulop (Medical University of South Carolina, USA)

Dr. Laetitia Koppe (University of Lyon, France)

Prof. Dr. Pang-Chui Shaw (Chinese University of Hong Kong, China)

Dr. Thérèse Malliavin (Unité de Bioinformatique Structurale, France)

Dr. Andrew D. Turner (Cefas, Food Safety Group, UK)

Dr. Michelle Dunstone (Monash University, Australia)

Dr. Marc Maresca (Aix Marseille Université, France)

Dr. Amparo Alfonso (University of Santiago of Compostela, Spain)

## 3. Organizers

MDPI (https://www.mdpi.com/)

*Toxins* (https://www.mdpi.com/journal/toxins)

## 4. Novel Plant, Animal, Insect, and Microbial Toxins

### 4.1. Genome Sequence Analysis and Insecticidal Characterization of Bacillus thuringiensis Bt-UNVM_94, a Strain Showing Dual Insecticidal Activity against Lepidopteran and Coleopteran Pests

PeraltaCecilia^1^SaukaDiego Herman^2^PérezMelisa^2^OncoMaría Ines^2^FiodorAngelika^3^CaballeroJavier^4^,^5^CaballeroPrimitivo^4^,^5^BerryColin^6^ValleEleodoro E. Del^7^PalmaLeopoldo^1^,^8^*1Centro de Investigaciones y Transferencia de Villa María (CIT-VM), Consejo Nacional de Investigaciones Científicas y Técnicas (CONICET), Universidad Nacional de Villa María, Villa María 5900, Argentina2Instituto Nacional de Tecnología Agropecuaria (INTA), Instituto de Microbiología y Zoología Agrícola (IMYZA), Castelar 1712, Argentina3Department of Biology, Institute of Microbiology, Bialystok University, 15097 Bialystok, Poland4Institute for Multidisciplinary Research in Applied Biology-IMAB, Universidad Pública de Navarra, 31192 Mutilva, Navarra, Spain5Bioinsectis SL, Avda Pamplona 123, 31421 Mutilva, Navarra, Spain6Cardiff School of Biosciences, Cardiff University, Park Place, Cardiff CF10 3AX, UK7Facultad de Ciencias Agrarias, Universidad Nacional del Litoral, Esperanza 3080, Argentina8Instituto Académico Pedagógico de Ciencias Básicas y Aplicadas (IAPCByA), Universidad Nacional de Villa María (UNVM), Villa María 5900, Argentina*Correspondence: palma.leopoldo@gmail.com

*Bacillus thuringiensis* (Bt) is a Gram-positive and spore-forming bacterium that synthesizes a wide diversity of proteins with insecticidal activity and that has demonstrated its potential and safety as a biocontrol agent for more than four decades. However, several susceptible insect species have been reported for evolving resistance, which demands screening for strains exhibiting novel insecticidal properties. In this work, we performed the genome sequence analysis and insecticidal characterization of a Bt strain designated Bt-UNVM_94, isolated from Argentina. Its genomic sequence harbors one coding sequence showing homology to the crystal protein Cry7Ga1, plus two others showing similarity to Mpp2Aa3 (ETX/Mtx2) protein and a putative mosquitocidal protein (NPP1). Cry7A and Cry7B are known to be distinctively active against some coleopteran and lepidopteran larvae, respectively. Spore–crystal mixtures used for SDS-PAGE analysis showed a band corresponding to the predicted size of Cry7Ga-like protein (~128 kDa). Bioassays performed also with spore–crystal mixtures exhibited dual toxicity, with 50% and 91% mortality against *Cydia pomonella* (Lepidoptera: Tortricidae) and *Anthonomus grandis* (Coleoptera: Curculionidae), respectively, representing, what we believe, the first insecticidal activity report for a Cry7Ga-like protein. Screenings of novel Bt strains may provide proteins with novel insecticidal properties that can be used to suppress insect resistance to the most used Bt crops in agriculture.

**Keywords:***Bacillus thuringiensis*; crystal proteins; Cry7 proteins; insecticidal activity; lepidopteran; coleopteran pests

**Key Contribution:** In this work, we report for the first time the insecticidal activity of a Cry7Ga-like protein from *Bacillus thuringiensis*.

### 4.2. The Insecticidal–Protein Repertory of 14 Xenorhabdus Strains Isolated from Argentina

PalmaLeopoldo^1^,^2^*CaballeroPrimitivo^3^,^4^BerryColin^5^FrizzoLaureano^6^ValleEleodoro E. Del^7^1Centro de Investigaciones y Transferencia de Villa María (CIT-VM), Consejo Nacional de Investigaciones Científicas y Técnicas (CONICET), Universidad Nacional de Villa María, Villa María 5900, Argentina2Instituto Académico Pedagógico de Ciencias Básicas y Aplicadas (IAPCByA), Universidad Nacional de Villa María (UNVM), Villa María 5900, Argentina3Institute for Multidisciplinary Research in Applied Biology-IMAB, Universidad Pública de Navarra, 31192 Mutilva, Navarra, Spain4Bioinsectis SL, Avda Pamplona 123, Mutilva, Navarra, Spain5Cardiff School of Biosciences, Cardiff University, Park Place, Cardiff CF10 3AX, UK6ICIVET (CONICET-UNL)—Departamento de Salud Pública, Facultad de Ciencias Veterinarias, Universidad Nacional del Litoral, Esperanza, Santa Fe, Argentina7ICiagro Litoral, Universidad Nacional del Litoral, CONICET, Facultad de Ciencias Agrarias; Esperanza CP3080, Argentina*Correspondence: palma.leopoldo@gmail.com

Entomopathogenic nematodes belonging to the genus *Steinernema* are able to infest and kill insect hosts in association with their resident entomopathogenic, symbiont bacteria in the Gram-negative genus *Xenorhabdus* (Enterobacteriaceae). However, only a few species of *Xenorhabdus* have been isolated from their hosts and their insecticidal properties reported. Here, we performed the genome sequence analysis of 14 *Xenorhabdus* strains isolated from *Steinernema* nematodes in Argentina, able to kill 6th instar *Galleria mellonella* (Lepidoptera: Pyralidae) larvae. The 14 draft genome sequences encoded a total of 110 putative insecticidal proteins (mostly Tc, Pra/Prb, and Mcf homologs) plus other virulence factors with similarity to putative nematocidal proteins and chitinases. The genome sequences of the strains Flor, 5, PSL, Reich, 42, Vera, M, 18, Cul, DI, 12, 38, 3, and ZM exhibited 4, 9, 2, 10, 9, 5, 7, 9, 10, 7, 3, 18, 8, and 8 putative insecticidal genes, respectively. Some strains carried their predicted insecticidal protein genes arranged into putative pathogenicity islands. Average nucleotide identity (ANI) calculations were also performed and allowed the identification of three strains that should be considered members of two novel *Xenorhabdus* genomospecies (strains PSL + Reich and strain 12). In this work, we provide a dual insight into the diversity of the species belonging to the *Xenorhabdus* genus and into their predicted insecticidal protein repertory, which is currently under investigation.

**Keywords:***Xenorhabdus* genus; Gram-negative entomopathogenic bacteria; insecticidal proteins; insect pests

**Key Contribution:** In this work, we report the genomic sequences and insecticidal–protein repertory of 14 *Xenorhabdus* strains isolated from Argentina.

### 4.3. Naturally Produced Beauvericins and Divergence of the BEAS Gene among Fusarium and Trichoderma Species

UrbaniakMonika^1^*WaśkiewiczAgnieszka^2^KoczykGrzegorz^3^BłaszczykLidia^4^UhligSilvio^5^StępieńŁukasz^1^1Plant-Pathogen Interaction Team, Department of Pathogen Genetics and Plant Resistance, Institute of Plant Genetics of the Polish Academy of Sciences, Strzeszyńska 34, 60-479 Poznań, Poland2Department of Chemistry, Poznan University of Life Sciences, Wojska Polskiego 75, 60-625 Poznań, Poland3Functional Evolution of Biological Systems Team, Department of Biometry and Bioinformatics, Institute of Plant Genetics of the Polish Academy of Sciences, Strzeszyńska 34, 60-479 Poznań, Poland4Plant Microbiome Structure and Function Team, Department of Pathogen Genetics and Plant Resistance, Institute of Plant Genetics of the Polish Academy of Sciences, Strzeszyńska 34, 60-479 Poznań, Poland5Norwegian Veterinary Institute, P.O. Box 750 Sentrum, 0106 Oslo, Norway*Correspondence: murb@igr.poznan.pl

Beauvericin (BEA) and its analogues are non-ribosomal cyclodepsipeptide mycotoxins produced by a wide range of fungal species, including saprotroph, plant, and insect pathogens, particularly belonging to *Fusarium*, *Trichoderma*, *Beauveria*, and *Isaria* genera. Most beauvericin analogues were described among *Beauveria* and *Isaria* genera as unnatural beauvericins by adding amino acid precursors to the growing media. The aim of the study was to find BEAs naturally synthesized by *Fusarium* species and tentatively determine their structures using mass spectrometry. Moreover, because of the unknown ability to produce beauvericin by *Trichoderma* fungi, we carried out quantitative analysis using ultra-high-performance liquid chromatography coupled with mass spectrometry (UPLC-MS). We also analyzed the polymorphism of the *BEAS* gene by sequencing partial *BEAS* regions from *Trichoderma* and *Fusarium* species. We screened five fungal cultures from the *Fusarium* genus cultivated on rice grain for the presence of the new natural beauvericins. The peptide sequence data of beauvericin analogues were established using MS/MS experiments as well as amino acid and hydroxy acid analysis following acid hydrolysis. Ten cyclodepsipeptide analogues described earlier were tentatively identified in the extract. In addition, two so far undescribed tyrosine-containing beauvericin analogues were tentatively identified in the cultures. Moreover, a quantitative analysis of beauvericin was performed using UPLC-MS in 11 *Trichoderm* and 6 *Fusarium* rice cultures. The phylogenic analyses of beauvericin synthase (*BEAS*) divergence were performed on the basis of sequenced PCR-amplified fragments from *Trichoderma* and *Fusarium* fungi and partial reference genes from the GenBank database (representing *Beauveri*a, *Fusarium*, and *Trichoderma* genera). This study demonstrates the high variability of naturally produced new types of beauvericins, such as tyrosine-containing analogues in *Fusarium* fungi. It also shows that fungi belonging to the *Trichoderma* genus possess the ability to produce beauvericin.

**Keywords:** beauvericin; *Fusarium*; *Trichoderma*; cyclodepsipeptide

### 4.4. Acute and Chronic In Vivo Toxicity of the Marine Toxin Palytoxin

Boente-JuncalAndreaRaposo-GarcíaSandraCostasCeliaLouzaoM CarmenOteroPazValeCarmen*BotanaLuisDepartamento de Farmacología, Farmacia y Tecnología Farmacéutica, Facultad de Veterinaria, Universidad de Santiago de Compostela, Lugo, Spain*Correspondence: mdelcarmen.vale@usc.es

Palytoxin (PLTX) is a marine toxin that nowadays is recognized amongst the most toxic compounds isolated from natural products. Originally, the toxin was only identified in a single tidal pool of the island of Maui (Hawaii). Currently, this compound is considered an emergent toxin in Europe, and its prevalence in continental European waters has increased during the past years. The high toxicity of palytoxin is related to the binding to Na^+^-K^+^ ATPase, converting this ubiquitously distributed enzyme in a permeant cation channel [1–3]. Several reports have shown that this toxin is responsible for human fatal intoxications, either after inhalation of toxin-containing marine aerosols or after ingestion of marine products contaminated by PLTX, such as crabs, groupers, mackerel, and parrotfish. So far, different groups have explored the acute oral toxicity of PLTX in mice; however, discrepancies in the PLTX source as well as in the monitoring time for the toxic effects have yielded controversial results. Although the presence of palytoxin in marine products is not yet currently regulated in Europe, the European Food Safety Authority (EFSA) expressed its opinion on PLTX toxicity and prompted the need to obtain more data regarding the in vivo toxicity of this compound [4]. Therefore, in this study, the acute and chronic toxicity of palytoxin was evaluated after oral administration of the toxin to mice, either in a single dose and in a follow-up period of 96 h or after chronic administration during a 28-day period. After chronic exposure of mice to the toxin, a median lethal dose (LD_50_) of 0.44 µg/kg of PLTX, much lower than that observed in the acute experiments, and a no-observed-adverse-effect level (NOAEL) of 0.03 µg/kg for repeated daily oral administration of PLTX were determined. Therefore, these data indicate a much higher chronic toxicity of PLTX and a lower NOAEL than those previously described in shorter treatment periods, remarking the need to further evaluate the potential teratogenic effects of this emerging marine toxin in mammals.

**Keywords:** palytoxin; toxicity; in vivo; food safety

**References**

1. Artigas, P.; Gadsby, D.C. Ion occlusion/deocclusion partial reactions in individual palytoxin-modified Na^+^/K^+^ pumps. *Ann. N. Y. Acad. Sci.* **2003**, *986*, 116–126.
2. Artigas, P.; Gadsby, D.C. Na^+^/K^+^-pump ligands modulate gating of palytoxin-induced ion channels. *Proc. Natl. Acad. Sci. USA* **2003**, *100*, 501–505.
3. Artigas, P.; Gadsby, D.C. Large diameter of palytoxin-induced Na^+^/K^+^ pump channels and modulation of palytoxin interaction by Na^+^/K^+^ pump ligands. *J. Gen. Physiol.* **2004**, *123*, 357–376.
4. EFSA. Panel on contaminants in the food chain (CONTAM). Scientific Opinion on marine biotoxins in shellfish–Palytoxin group. *EFSA J.* **2009**, *7*, 1393.

### 4.5. Hemolytic Activity of Venoms of the Water Shrew Neomys fodiens and the Common Shrew Sorex araneus

KowalskiKrzysztof^1^*MarciniakPaweł^2^RychlikLeszek^3^1Department of Vertebrate Zoology and Ecology, Institute of Biology, Faculty of Biological and Veterinary Sciences, Nicolaus Copernicus University in Toruń, Toruń, Poland2Department of Animal Physiology and Development, Institute of Experimental Biology, Faculty of Biology, Adam Mickiewicz University, Uniwersytetu Poznańskiego 6, 61-614 Poznań, Poland3Department of Systematic Zoology, Institute of Environmental Biology, Faculty of Biology, Adam Mickiewicz University, Uniwersytetu Poznańskiego 6, 61-614 Poznań, Poland*Correspondence: k.kowalski@umk.pl

Venomous mammals are rare, and their venoms have not been comprehensively investigated. Among shrews, only venoms of the short-tailed shrew Blarina brevicauda and the Eurasian water shrew *Neomys fodiens* have been characterized thus far. *Neomys fodiens* employs its venom to hunt on larger prey and store it in a comatose state. Recently, the potent paralytic activity of its venom has been confirmed. Here, we assayed the hemolytic effects of crude extracts of the salivary glands of N. fodiens and the common shrew Sorex araneus in the red blood cells of frogs. Toxins present in saliva were identified by high-performance liquid chromatography coupled with tandem mass spectrometry. For both shrew species, we found significant concentration-dependent effects of the venom on hemolysis in erythrocytes, evaluated as hemoglobin release. Hemolytic effects of N. fodiens saliva were stronger than those produced by *S. araneus*. We identified four toxins in N. fodiens venom and five in the saliva of *S. araneus*. Some of them are likely to produce hemolysis in the frog’s erythrocytes. Our results show that shrew venoms, in addition to having potent paralytic properties, also possess hemolytic activity that may allow them to hunt larger prey such as frogs. Additionally, because *S. araneus* saliva exhibits toxic activity, we propose to add the common shrew to the list of venomous mammals.

**Keywords:** eulipotyphlans; hemolysis; mammalian venom; natural toxins; shrews; toxic saliva

### 4.6. Cloning and Expression of a Hemocyanin Isolated from the Centipede Cryptops iheringi

CruzKariny^1^*NishiyamaMilton Y.Jr.^2^AzevedoInácio de Loiola Meirelles Junqueira de^2^MagalhãesGeraldo^1^*Shimokawa-FalcãoLhiri Hanna Alves De Lucca^1^*1Immunopathology Laboratory, Instituto Butantan, São Paulo, Brazil2Special Laboratory to Applied Toxinology, Instituto Butantan, São Paulo, Brazil*Correspondence: kariny.cruz@butantan.gov.br (K.C.); geraldo.magalhaes@butantan.gov.br (G.M.); lhiri.lucca@butantan.gov.br (L.H.A.D.L.S.-F.)

Among the centipedes of the chilopode Myriapoda class, the Cryptops genus is one of the most associated with accidents in humans in the metropolitan region of the state of São Paulo. To date, there is no study in the literature about *Cryptops iheringi* toxins. Thus, in this work, transcriptomic analysis of the C. iheringi venom gland was performed to obtain a profile of the toxins of this species. In addition, the crude venom was subjected to mass spectrometry analysis to establish an association between unknown sequences. These approaches for the construction of a general profile of the venom gland expression of this species led to the identification of a hemocyanin (Hc) subunit. Hemocyanins are copper-containing respiratory proteins that occur in the hemolymph of many arthropod species. Here, we report the presence of Hc in the chilopode Myriapoda C. iheringi. Such respiratory proteins have long been considered unnecessary in Myriapoda due to its tracheal systems. These respiratory proteins are potent immunogens, which induce the synthesis of large amounts of specific antibodies. Studies have pointed out their interaction with polymorphonuclear monocytes and lymphocytes, and in vitro tests have shown a potential anticancer activit, with in vitro significant inhibition of the growth of cancerous strains of the breast, pancreas, and prostate. Currently, scientific data are mostly limited to the study of native Hc of M. crenulata molluscs; therefore, the biotechnological potential of Hcs isolated from centipedes is still unexplored. Herein, the Hc sequence that was present in both proteome and transcriptome analysis has a signal peptide and a 76 kDa range. The Hc subunit sequence was synthesized with codon optimization for bacteria expression and the protein expressed as inclusion bodies. Refolding attempts provided soluble forms of Hc. At the moment, efforts to access its biological activities are being carried out.

**Keywords:***Cryptops iheringi*; centipede; hemocyanin

### 4.7. Analysis of the Pimelea Toxin Simplexin for the Development of a Cattle Microbial Probiotic

LohZhi Hung^1^*HungerfordNatasha L.^1^OuwerkerkDiane^1^,^2^KlieveAthol V.^1^FletcherMary T.^1^1Queensland Alliance for Agriculture and Food Innovation (QAAFI), The University of Queensland, Health and Food Sciences Precinct, Coopers Plains, QLD 4108, Australia2Agri-Science Queensland, Department of Agriculture and Fisheries (QDAF), Ecosciences Precinct, Dutton Park, QLD 4102, Australia*Correspondence: zhihung.loh@uq.edu.au

Pimelea poisoning of cattle (also known as St. George or Marree disease) is a poisoning unique to Australia and caused by inadvertent grazing of native Pimelea within pastures. The toxin responsible for the poisoning was previously isolated and identified as the novel diterpenoid orthoester simplexin, but no effective treatments for poisoned animals exist. A previous feeding trial reported that cattle fed daily with increasing low doses of simplexin showed reduced poisoning symptoms over time, which suggested cattle develop resistance against the toxin, potentially via by the adaptation of rumen microorganisms. To date, there are no reports on simplexin degradation by rumen microorganisms. This study aims to develop a microbial probiotic derived from the rumen fluid of field-exposed animals that is capable of detoxifying simplexin, thus allowing cattle to consume Pimelea with less adverse effects. Investigations are ongoing to identify rumen bacteria able to hydrolyze simplexin in in vitro mixed rumen-based anaerobic fermentations fed daily with Pimelea plant species (*P. trichostachya*) and to assess isolated rumen bacteria in in vitro incubation trials. Simplexin levels in both studies were analyzed by ultra-high-performance liquid chromatography coupled with high-resolution, accurate tandem mass spectrometry (UPLC-MS/MS), which allows simplexin quantification at ppb concentrations (ng/mL) on a Thermo Scientific Q-Exactive Orbitrap mass spectrometer. Results to date showed decreases in simplexin levels, suggestive of simplexin detoxification by rumen microorganisms. Simplexin acid hydrolysis studies were also performed to create a metabolite database to aid in future elucidation of potential simplexin degradation pathways. UPLC-MS/MS analysis based on predicted molecular formulae enabled identification of three hydrolyzed simplexin products, which also shared several fragment ions with simplexin. Future studies will include the identification and characterization of simplexin metabolites from fermentation and incubation trials in which simplexin levels indicate that degradation has occurred.

**Keywords:** plant toxins; probiotic; rumen microorganisms; metabolism; degradation

## 5. Mechanism of Action and/or Pathophysiology of Toxins

### 5.1. The Effect of a Hydrogen Peroxide Preparation with Silver Ions on the Qualitative Traits of Table Eggs and on Reducing Mycotoxin Biosynthesis

TomczykŁukasz^1^*SzablewskiTomasz^1^Stuper-SzablewskaKinga^2^BiadałaAgata^1^KoniecznyPiotr^1^NowaczewskiSebastian^3^Cegielska-RadziejewskaRenata^1^1Department of Food Safety and Quality Management, Poznan University of Life Sciences, Wojska Polskiego 31, 60-624 Poznan, Poland2Department of Chemistry, Poznan University of Life Sciences, Wojska Polskiego 75, 60-625 Poznań, Poland3Department of Animal Breeding and Product Quality Assessment, Poznan University of Life Sciences, 60-637 Poznan, Poland*Correspondence: tomczyk@up.poznan.pl

The quality and safety of raw materials and food products are inextricably linked. Table eggs are subject to special monitoring due to microbial hazards. So far, bacterial hazards have mostly been monitored. However, the latest reports have pointed to a threat that has not been considered for table eggs. Microfungi can grow on the surface of eggshells and penetrate into the egg content. Therefore, it is necessary to improve the microbiological state of the eggshell surface, which will guarantee the safety of egg consumption and slow down spoilage. The aim of the study was to examine how the sanitation of eggs with a hydrogen peroxide preparation containing silver ions affects the dynamics of growth of microfungi and the biosynthesis of mycotoxins during egg storage. The research results showed that H_2_O_2_ with silver ions is effective against microfungi and simultaneously limits the biosynthesis of mycotoxins. Egg sanitation treatment with a solution of hydrogen peroxide and silver ions reduced the count of microfungi, which stopped growing after one week of storage. The effectiveness of much lower concentrations of the preparation against these fungi may have been caused by the content of silver ions. There was a smaller decrease in the Haugh unit value in eggs sanitized with hydrogen peroxide and silver ions in the final period of storage. This means that the eggs lost freshness less dynamically. The research results showed that the treatment of eggs with the H_2_O_2_ preparation with silver ions slowed down their spoilage processes and effectively reduced their content of microfungi and mycotoxins.

**Keywords:** silver ions; hydrogen peroxide; eggs; microfungi; mycotoxins

### 5.2. The Enteric Nerve System as a Target of Regulated and Emerging Food-Associated Mycotoxins

DąbrowskiMichał^1^OlleikHamza^2^KadriAmine^2^CampsValérie^2^PerrierJosette^2^PintonPhilippe^3^OswaldIsabelle P.^3^ZielonkaŁukasz^1^MarescaMarc^2^*1Department of Veterinary Prevention and Feed Hygiene, Faculty of Veterinary Medicine, University of Warmia and Mazury in Olsztyn, Oczapowski Str. 13/29, 10-718 Olsztyn, Poland2Aix Marseille University, CNRS, Centrale Marseille, iSm2, 13397 Marseille, France3Toxalim, Research Center in Food Toxicology, Université de Toulouse, INRAE, ENVT, INP- PURPAN, UPS, F-31027 Toulouse, France*Correspondence: m.maresca@univ-amu.fr

Food and feed are frequently contaminated by numerous regulated and emerging mycotoxins. Humans and animals are thus exposed daily to mycotoxins through the oral route, making the gut the first and the more exposed tissue. Although many studies have evaluated and demonstrated the impact of mycotoxins on intestinal epithelial cells (IECs) and on brain cells, surprisingly only few studies have investigated their impact on cells of the enteric nerve system (ENS). In the present work, we measured the impact of major regulated and emerging mycotoxins (18 mycotoxins in total) on the proliferation and viability of normal rat enteric glial cells (EGCs) in vitro. Of the 18 mycotoxins tested, 12 were found toxic, with anti-proliferative and/or cytotoxic effects observed at doses ranging from 0.19 to 118 µM and 0.4 to 59.59 µM, respectively. It can be concluded that alterations in EGCs caused by at least some mycotoxins may participate in their global impact on the gut and the full organism.

**Keywords:** food safety; food contaminants; mycotoxins; emerging mycotoxins; enteric nerve system; enteric glial cells; cyclohexadepsipeptide

**Key Contribution:** Mycotoxins are able to affect the proliferation and viability of enteric glial cells, suggesting the implication of alteration in the ENS in mycotoxicosis.

### 5.3. Effects of Synthetic Ciguatoxin CTX3C and 44-Methylgambierone (MTX3) on Voltage-Gated Sodium Channels and Their In Vivo Toxicity

Boente-JuncalAndreaRaposo-GarcíaSandraCostasCeliaLouzaoM CarmenOteroPazValeCarmen*BotanaLuisDepartamento de Farmacología, Farmacia y Tecnología Farmacéutica, Facultad de Veterinaria, Universidad de Santiago de Compostela, Lugo, Spain*Correspondence: mdelcarmen.vale@usc.es

*Gambierdiscus* species are marine dinoflagellates producers of toxins causative of a widespread human illness known as ciguatera fish poisoning (CFP), which includes gastrointestinal, neurological, and cardiovascular symptoms. Blooms of these dinoflagellates have expanded worldwide, reaching even European coasts. In fact, the presence of *Gambierdiscus* species and the related toxins and CFP intoxications have been repetitively identified in Europe during the past decades, especially in the Canary Islands [1,2] and Madeira [3]. In addition to ciguatoxins, which can cause long-term neurological sequela in humans as a consequence of their permanent activation voltage-gated sodium channels [4–6], the structure of an additional ciguatoxin-related toxin named 44-methylgambierone (MTX3) has been recently elucidated [7]. Initial studies on the biological activity of 44-methylgambierone described an effect similar to that of the synthetic ciguatoxin CTX3C, although of much lower potency [7]. With the aim of further exploring the relative toxicities and activities of these compounds, additional experiments were performed. First, the neurotoxic effect of CTX3C and MTX3 was evaluated using a human neuronal cell model based on the incubation of SH-SY5Y with ouabain and veratridine, together with ciguatoxin or ciguatoxin-like compounds, to evaluate their in vitro toxic potency [8]. Our data illustrate that CTX3C aggravates ouabain and veratridine neurotoxicity, but 44-methylgambierone did not resemble this effect. Additionally, while CTX3C at nanomolar concentrations hyperpolarized the activation of voltage-gated sodium channels and decreased the current amplitude, 44-methylgambierone did not affect sodium currents. Moreover, oral chronic toxicity studies using daily CTX3 concentrations of 10, 32, and 100 ng/kg or MTX3 at 550 or 1760 ng/kg and an observation period of 28 days did not show behavioral or biochemical alterations during treatment. Based on in vitro and in vivo results, the ciguatoxin-related compound 44-methylgambierone, recently identified in *Gambierdiscus* extracts, is less potent than CTX3C and thus indicates that the effect on human CFP symptoms may also be minor.

**Keywords:** ciguatoxin; 44-methylgambierone (MTX3); ciguatera fish poisoning; neurotoxicity; voltage-gated sodium channel; chronic toxicity

**References**

1. Estevez, P.; Sibat, M.; Leão-Martins, J.M.; Tudó, A.; Rambla-Alegre, M.; Aligizaki, K.; Diogène, J.; Gago-Martinez, A.; Hess, P. Use of Mass Spectrometry to Determine the Diversity of Toxins Produced by Gambierdiscus and Fukuyoa Species from Balearic Islands and Crete (Mediterranean Sea) and the Canary Islands (Northeast Atlantic). *Toxins* **2020**, *12*, 305.
2. Perez-Arellano, J.L.; Luzardo, O.P.; Perez Brito, A.; Hernandez Cabrera, M.; Zumbado, M.; Carranza, C.; Angel-Moreno, A.; Dickey, R.W.; Boada, L.D. Ciguatera fish poisoning, Canary Islands. *Emerg. Infect. Dis.* **2005**, *11*, 1981–1982.
3. Otero, P.; Pérez, S.; Alfonso, A.; Vale, C.; Rodríguez, P.; Gouveia, N.N.; Gouveia, N.; Delgado, J.; Vale, P.; Hirama, M., et al. First toxin profile of ciguateric fish in Madeira Arquipelago (Europe). *Anal. Chem.* **2010**, *82*, 6032–6039.
4. Martín, V.; Vale, C.; Hirama, M.; Yamashita, S.; Rubiolo, J.A.; Vieytes, M.R.; Botana, L.M. Synthetic ciguatoxin CTX 3C induces a rapid imbalance in neuronal excitability. *Chem. Res. Toxicol.* **2015**, *28*, 1095–1108.
5. Martín, V.; Vale, C.; Rubiolo, J.A.; Roel, M.; Hirama, M.; Yamashita, S.; Vieytes, M.R.; Botana, L.M. Chronic ciguatoxin treatment induces synaptic scaling through voltage gated sodium channels in cortical neurons. *Chem. Res. Toxicol.* **2015**, *28*, 1109–1119.
6. Vale, C.; Antero, A.; Martín, V. Pharmacology of ciguatoxins. In *Phycotoxins, Chemistry and Biochemistry*; Alfonso, LBAA, Ed.; Wiley Blackwell: Hoboken, NJ, USA, 2015; pp. 23–48.
7. Boente-Juncal, A.; Álvarez, M.; Antelo, Á.; Rodríguez, I.; Calabro, K.; Vale, C.; Thomas, O.P.; Botana, L.M. Structure Elucidation and Biological Evaluation of Maitotoxin-3, a Homologue of Gambierone, from Gambierdiscus belizeanus. *Toxins* **2019**, *11*, 79.
8. Coccini, T.; Caloni, F.; De Simone, U. Human neuronal cell based assay: A new in vitro model for toxicity evaluation of ciguatoxin. *Environ. Toxicol. Pharmacol.* **2017**, *52*, 200–213.

### 5.4. Oral Chronic Toxicity of the Marine Toxin Tetrodotoxin

Raposo-GarcíaSandraBoente-JuncalAndreaCostasCeliaLouzaoM CarmenOteroPazValeCarmen*BotanaLuisDepartamento de Farmacología, Farmacia y Tecnología Farmacéutica, Facultad de Veterinaria, Universidad de Santiago de Compostela, Lugo, Spain*Correspondence: mdelcarmen.vale@usc.es

Tetrodotoxin (TTX) is a toxic compound responsible for human intoxication after ingestion of contaminated fishery products. Although TTX was initially associated mainly with human fatalities occurring in Asiatic countries [1], nowadays it has expanded to other regions, including European countries. In Europe, the first non-fatal human intoxication by TTX was reported more than 10 years ago after the ingestion of a *Charonia lampas* trumpet shell captured in the Portuguese coast and commercialized in Spain [2]. Since then, during the past decade, the presence of the TTX-containing pufferfish *Lagocephalus sceleratus* has been reported in European coasts, mainly in the Mediterranean Sea [3,4], with some fish tissues containing TTX amounts as high as 2 mg/kg [5]. Moreover, an increasing concern regarding food safety has been raised after the detection of TTX in mussels, oysters, and clams harvested in the UK, Greece, the Netherlands [6–8], and Spain. The current European legislation on marine toxins does not yet regulate the levels of TTX in fishery products, and nowadays, the presence of the toxin is only regularly monitored in the Netherlands, although the European Food Safety Authority (EFSA) has recommended the level of 44 µg/kg TTX for routine monitoring, since, at this dose, no adverse effects were observed in humans. Considering initial data on the acute oral toxicity of TTX and in view of the EFSA’s opinion remarking the need for additional chronic toxicity studies to further reduce the uncertainty of the likely future toxin regulation, in this work, the oral chronic toxicity of TTX using doses of 25, 44, 75, and 125 µg/kg and an observation period of 28 days was explored in female mice using protocols internationally validated to test the toxicity of chemicals. The data presented here indicated that 25 and 44 µg/kg of TTX did not cause either blood biochemical or behavioral alterations in mice, while at the dose of 125 µg/kg, kidney and heart alterations were observed under electron microscopy analysis. Therefore, the data presented here indicate that the safe TTX dose proposed by the EFSA is low enough to prevent human adverse effects, while caution should be taken in the presence of higher TTX doses.

**Keywords:** tetrodotoxin; marine toxins; in vivo toxicity; risk assessment

**References**

1. Bane, V.; Lehane, M.; Dikshit, M.; O’Riordan, A.; Furey, A. Tetrodotoxin: chemistry, toxicity, source, distribution and detection. *Toxins* **2014**, *6*, 693–755.
2. Rodriguez, P.; Alfonso, A.; Vale, C.; Alfonso, C.; Vale, P.; Tellez, A.; Botana, L.M. First Toxicity Report of Tetrodotoxin and 5,6,11-TrideoxyTTX in the Trumpet Shell Charonia lampas lampas in Europe. *Anal. Chem.* **2008**, *80*, 5622–5629.
3. Rambla-Alegre, M.; Reverte, L.; Del Rio, V.; de la Iglesia, P.; Palacios, O.; Flores, C.; Caixach, J.; Campbell, K.; Elliott, C.T.; Izquierdo-Munoz, A., et al. Evaluation of tetrodotoxins in puffer fish caught along the Mediterranean coast of Spain. Toxin profile of Lagocephalus sceleratus. *Environ. Res.* **2017**, *158*, 1–6.
4. Katikou, P.; Georgantelis, D.; Sinouris, N.; Petsi, A.; Fotaras, T. First report on toxicity assessment of the Lessepsian migrant pufferfish Lagocephalus sceleratus (Gmelin, 1789) from European waters (Aegean Sea, Greece). *Toxicon* **2009**, *54*, 50–55.
5. Leonardo, S.; Kiparissis, S.; Rambla-Alegre, M.; Almarza, S.; Roque, A.; Andree, K.B.; Christidis, A.; Flores, C.; Caixach, J.; Campbell, K.; et al. Detection of tetrodotoxins in juvenile pufferfish Lagocephalus sceleratus (Gmelin, 1789) from the North Aegean Sea (Greece) by an electrochemical magnetic bead-based immunosensing tool. *Food Chem.* **2019**, *290*, 255–262.
6. Katikou, P. Public Health Risks Associated with Tetrodotoxin and Its Analogues in European Waters: Recent Advances after The EFSA Scientific Opinion. *Toxins* **2019**, *11*, 240.
7. Turner, A.D.; Powell, A.; Schofield, A.; Lees, D.N.; Baker-Austin, C. Detection of the pufferfish toxin tetrodotoxin in European bivalves, England, 2013 to 2014. *Eurosurveillance* **2015**, *20*, 21009.
8. Vlamis, A.; Katikou, P.; Rodriguez, I.; Rey, V.; Alfonso, A.; Papazachariou, A.; Zacharaki, T.; Botana, A.M.; Botana, L.M. First Detection of Tetrodotoxin in Greek Shellfish by UPLC-MS/MS Potentially Linked to the Presence of the Dinoflagellate Prorocentrum minimum. *Toxins* **2015**, *7*, 1779–1807.

### 5.5. The Regulatory Mechanisms of Cyanotoxin β-N-Methylamino-L-Alanine (BMAA) Action on the Key Cellular Processes in Diazotrophic Cyanobacteria

KoksharovaOlga^1^*ButenkoIvan O^2^PobegutsOlga V^2^SafronovaNina A^1^GovorunVadim M^2^1Lomonosov Moscow State University, Belozersky Institute of Physico-Chemical Biology, Leninskie Gory, 1-40, Moscow 119991, Russia2Scientific-Research Institute of Physical-Chemical Medicine, Moscow 119435, Russia*Correspondence: oa-koksharova@rambler.ru

The non-proteinogenic neurotoxic amino acid β-N-methylamino-L-alanine (BMAA) is a bioactive molecule synthesized by various phytoplankton species, such as cyanobacteria, diatoms, and dinoflagellates, and is known to be a causative agent of human neurodegenerative diseases. The ability of different microalgae to synthesize BMAA may be an indicator of the importance of this molecule in the interaction of phytoplankton organisms in nature. We were interested in the question of what kinds of mechanisms underline BMAA’s action on cyanobacterial cells under different nitrogen supply conditions. To answer this question, we performed molecular studies using a model cyanobacterial strain *Nostoc * (*Anabaena*) sp. PCC 7120. We experimentally showed that the action of BMAA on nitrogen-fixing filamentous cyanobacteria changes the nitrogen–carbon balance regulation and differs under nitrogen-starving and nitrogen-replete conditions. The primary main targets of BMAA’s action in cyanobacteria cells are, apparently, metabolic processes, such as nitrogen fixation, photosynthesis, carbon fixation, and different biosynthetic processes, the regulation of which involves 2-oxyglutarate and glutamate. Our proteomic study demonstrated that under BMAA treatment, the most significant difference lies in the expression change of a key nitrogen regulatory protein PII. This protein is downregulated in nitrogen-starving conditions, and it is upregulated in nitrogen-replete conditions in the presence of BMAA. This could be the main reason behind a specific regulatory effect on heterocyst formation and heterocyst- and nitrogenase-related gene expression that this amino acid causes in *Nostoc* sp. PCC 7120. Due to the fact that all metabolic processes are interconnected and well balanced in cyanobacteria cells, the disturbance in nitrogen metabolism leads to changes in carbon metabolism and photosynthesis. This explains the severe changes of CO_2_ fixation proteins and photosystem reaction center proteins that were found in our proteomics studies. BMAA addition leads to disorder in both amino acid synthesis and purine synthesis, as well as disturbing DNA transcription and protein translation. Finally, many enzymes of oxidative stress, chaperones, and SOS response proteins are upregulated under such metabolic stress conditions. Therefore, we can conclude that the disbalance in energy and metabolite amounts leads to severe intracellular stress that induces the upregulation of stress-activated proteins, such as starvation-inducible DNA-binding protein, stress response enzymes, proteases, and SOS response and DNA repair enzymes. It can be hypothesized that BMAA could be used by phytoplankton representatives (cyanobacteria, diatom, dinoflagelates) as a possible allelopatic tool to control cyanobacteria cell populations during their competition for nitrogen and other resources.

**Keywords:** BMAA; nitrogen fixation; nitrogenase; heterocyst differentiation; NtcA; PII protein; DNA repair; oxidative stress response; photosynthesis starvation; toxic molecule

### 5.6. In Silico Analysis of Short Linear Motifs Present in Snake Venom Phospholipases

PeggionCaterina^1^TonelloFiorella^2^*1Department of Biomedical Sciences, University of Padua, Padua, Italy2Institute of Neuroscience of the National Research Council, Padua, Italy*Correspondence: fiorella.tonello@cnr.it

Phospholipases A2 (PLA2s) are important constituents of snake venom that, depending on their amino acidic composition, possess several toxic properties, the main ones being neurotoxicity, myotoxicity, and impairing of hemostasis. They are proteins of about 120 amino acids, having a structure conserved since basal metazoa and similar to that of mammalian secretory PLA2s. Some snake venom PLA2s are heteromultimers, while others are monomers or homodimers. In this work, we analyzed the sequence alignment of monomeric or homodimeric snake venom PLA2s grouped according to their myotoxic and neurotoxic properties, and we compared this alignment with that of the most similar mammalian secretory PLA2s. We found short linear motifs present in three regions of secretory PLA2s that can play a role in their toxic and physiological functions. This work suggests important molecular interactions of secretory PLA2s that can focus and shorten the experimental work of characterization of the mechanism of action of these proteins.

**Keywords:** snake venom phospholipases A2; neurotoxins; myotoxins

### 5.7. Profiling of Signaling Pathways in Human Dermal Blood and Lymphatic Endothelial Cells Induced by Snake Venom Cysteine-Rich Secretory Protein (svCRiSP) from North American Snakes

SuntravatMontamas^1^,^2^*SanchezOscar^1^ReyesArmando^1^CiriloAbcde^1^OcheltreeJack S.^1^GalanJacob A.^1^,^2^SalazarEmelyn^1^DaviesPeter^3^SanchezElda E.^1^,^2^1National Natural Toxins Research Center (NNTRC), Texas A&M University-Kingsville, MSC 224, 975 West Avenue B, Kingsville, TX 78363, USA2Department of Chemistry, Texas A&M University-Kingsville, MSC 161, Kingsville, TX 78363, USA3Institute of Biosciences and Technology, Texas A&M University, Houston, TX, USA*Correspondence: montamas.suntravat@tamuk.edu

Snake venom cysteine-rich secretory proteins (svCRiSPs) are important components of the venom of many snake species. Little is known about the contribution that they make to the local pathophysiology of snakebites. We investigated the role of svCRiSPs from the most medically significant species of North American snakes (*Crotalus atrox*, *C. adamanteus*, *C. scutulatus scutulatus*, *C. horridus*, and *Agkistrodon piscivorus*), focusing on the cellular and molecular mechanisms. We evaluated the biological activities of svCRiSPs (Catrox-CRiSP, Cada-CRiSP, Css-CRiSP, Chor-CRiSP, and App-CRiSP) by using both in vitro assays of human dermal lymphatic endothelial cell (HDLEC) and blood endothelial cell (HDBEC) permeability and in vivo Miles assay. Of all the CRiSPs tested, Css-CRiSP and App-CRiSP displayed the highest increase in permeability compared to other crotaline CRiSPs. We initially screened the changes in protein expression and phosphorylation in HDLECs and HDBECs after treatment with Css-CRiSP and App-CRiSP using reverse-phase protein arrays (RPPAs). Studies are ongoing for identifying the key signaling that is involved in endothelial permeability after treatment with App-CRiSP and Css-CRiSP.

**Keywords:** signaling pathway; reverse-phase protein arrays (RPPAs); snake venom cysteine-rich secretory proteins (svCRiSPs); endothelial permeability; North American snakes

**Key Contribution:** Knowledge gained from these studies provides insights into the molecular mechanisms that underlie the effects of svCRiSPs on vascular function and contributes to a new level of understanding of the pathophysiology of snakebites.

### 5.8. Biological Characterization of a Kunitz-Type Inhibitor from Malaysian King Cobra (Ophiophagus hannah) Venom

SalazarEmelyn^1^RodriguezKassandra^1^SuntravatMontamas^1^,^2^SánchezElda E.^1^,^2^*1National Natural Toxins Research Center (NNTRC), Texas A&M University—Kingsville, 975 W. Avenue B, Kingsville, TX 78363, USA2Department of Chemistry, Texas A&M University—Kingsville, 700 University Blvd, MSC 161, Kingsville, TX 78363, USA*Correspondence: elda.sanchez@tamuk.edu

Kunitz-type inhibitors (KTIs) are proteins that bear homology to the bovine pancreatic trypsin inhibitor (BPTI) and exhibit a wide variety of biological activities, including inhibition of various proteases, interference with hemostasis, and inflammation, showing their functional diversity. KTIs have been isolated and identified as either chymotrypsin or trypsin inhibitors. This study aims to isolate and further characterize the pharmacological properties of a KTI from the Malaysian king cobra (*Ophiophagus hannah*) venom. The inhibitory effect on serine protease activity was determined using chromogenic substrates. The whole venom was fractionated by size-exclusion HPLC, and the isolated peaks were identified with N-terminal sequencing. The fractions were then incubated with plasmin at different times, and the inhibition of its biological activities was tested. The whole venom reduced the trypsin activity toward its chromogenic substrate. After size-exclusion chromatography, 13 fractions were isolated. After testing their effects on plasmin activity, we found that F10 showed the most remarkable effect, preventing fibrinolytic activity on fibrin plates and partially inhibiting fibrinogenolytic activity. These characterization studies will elucidate the biomedically relevant pharmacological properties intrinsic to a KTI from king cobra venom, leading to their potential use for biomedical applications.

**Keywords:** king cobra; Kunitz-type inhibitor; hemostasis; fibrinolysis; plasmin inhibitor

**Key Contribution:** A KTI named OH-TCI from the Malaysian king cobra venom was partially purified and characterized. In addition to its inhibitory activity toward trypsin, here we showed for the first time that OH-TCI could inhibit the activity of a serine protease from the hemostatic system, plasmin, toward its biological substrates fibrin and fibrinogen.

### 5.9. Blistering in Bothrops atrox Envenomings: Evidence of Antivenom and Inflammatory Factors at the Bite Site

GimenesSarah Natalie Cirilo^1^*SachettJacqueline^2^ColombiniMônica^1^Freitas-de-SousaLuciana^1^IbiapinaHiochelson Najibe dos Santos^2^CostaAllyson Guimarães^2^SantanaMonique^2^ParkJeong-Jin^3^ShermanNicholas^3^FerreiraLuiz Carlos de Lima^4^WenFan Hui^1^MonteiroWuelton Marcelo^2^SilvaAna Maria Moura da^1^FoxJay W^4^1Laboratório de Imunopatologia, Instituto Butantan, São Paulo SP, Brazil2Escola Superior de Ciências da Saúde, Universidade do Estado do Amazonas, Manaus, AM, Brazil3University of Virginia, Charlottesville, VA, USA4Departamento de Ensino e Pesquisa, Fundação de Dermatologia Alfredo da Matta, Manaus, AM, Brazil*Correspondence: sarah.gimenes@butantan.gov.br

In the Brazilian Amazon, there is a significant occurrence of snakebites, predominated by *Bothrops atrox*. Tissue damage is one of the hallmarks of *B. atrox* envenoming. Interestingly, many snakebite patients have delayed onset of blistering with a concomitant increase in the risk of infections. We hypothesize that blister fluid may represent a window into the pathophysiology of injured tissues. In this study, we examined blister fluid by proteomics from five patients hospitalized with *B. atrox* envenomation, who were successfully treated with antivenom, with no long-lasting effects or morbidities. The proteomic data of the blister fluid correlated with previous blister fluid studies showing the presence of DAMPs and immunomodulators. The blister composition was observed to be similar among the patients regardless of the clinical severity of envenomation. An unprecedented additional finding was that we identified venom and antivenom proteins in the bite site by ELISA. The venom was quantified in the fluid a significant time after envenommation (up to 135 h), suggesting slow clearance of the venom at the site of the bite, which might have an influence on local tissue well after the time of envenomation. Antibodies from the administered antivenom identified in the blister fluid were shown capable of binding venom proteins, by Western blotting. Thus, blister fluid antibodies should be capable of neutralizing any venom components in the fluid. Taken together, these findings suggest that although blistering is a delayed phenomenon of envenomation, its likely pathophysiological origins occur in advance of antivenom administration and venom neutralization at the site of envenomation and it continues despite the eventual neutralization of venom. This evidence confirms previous reports that the early events in envenomation pathophysiology give rise to endogenous factors that, over time, contribute to the development of blisters that are not attenuated even by prompt antivenom administration.

**Keywords:***Bothrops atrox*; blister; local damage; snake venom; antivenom; DAMPs; snakebite

### 5.10. Functional Characterization of a Novel Recombinant Cysteine-Rich Secretory Protein (rCRiSP) from Crotalus oreganus Helleri

SanchezOscar^1^CastilloEmelyn Salazar^1^GalanJacob A.^1^SanchezElda E.^1^,^2^SuntravatMontamas^1^1National Natural Toxins Research Center, Texas A&M University-Kingsville, Kingsville, TX, USA2Department of Chemistry, Texas A&M University-Kingsville, MSC 161, Kingsville, TX, USA*Correspondence: montamas.suntravat@tamuk.edu

Snake venom is a highly complex and diverse cocktail of different proteins and peptides that cause a wide range of biological disturbances in an envenomated victim. While many snake venom toxins have been comprehensively characterized, other toxins such as cysteine-rich secretory proteins (CRiSPs) remain largely unexplored. CRiSPs are ubiquitous non-enzymatic toxins found in many species of snakes worldwide. Several CRiSPs isolated from Asian and Australian snake venoms have been shown to inhibit ion channel/smooth muscle contraction. We recently reported that hellerin, a snake venom cysteine-rich secretory protein (svCRiSP) that we isolated from the venom of the Southern Pacific rattlesnake, Crotalus oreganus helleri, directly increases vascular permeability in vivo and in vitro. These observations may be parallel to Bj-CRP’s local effects, a CRiSP isolated from the venom of Bothrops jararaca, that has been shown to induce profound inflammatory responses in local tissue through the recruitment of neutrophils and the production of IL-6. To shed new light on svCRiSPs’ molecular targets and inflammatory responses, a recombinant CRiSP from C. o. helleri (named rHellerin) was cloned and tested for vascular and cellular permeability and pro-inflammatory responses. rHellerin was able to induce vascular leakage in vivo and cellular permeability similar to that of native CRiSP. rHellerin was also able to induce increased production of the cytokines IL-8 and IL-6 in human blood and lymphatic endothelial cells. These findings can provide a straightforward method of obtaining biologically viable svCRiSPs identical to the native form, which can accelerate research into further understanding the molecular biology of svCRiSPs by elucidating functionally active residues and subsequent molecular targets/interactions. rHellerin can have potential in the development of new therapeutic strategies to prevent death and disability from snakebites.

**Keywords:** recombinant protein; rHellerin; inflammatory responses; cysteine-rich secretory proteins (CRiSPs)

### 5.11. Evaluation of In Vitro Muscle Regeneration after Myonecrosis Induced by Bothrops alternatus and Bothrops diporus Venoms from Northeastern Argentina

FuscoLuciano SebastiánVeldeAndrea Van deLeivaLaura CBustilloSoledad*IQUIBA CONICET, Grupo de Investigaciones Biológicas y Moleculares (GIBM), Universidad Nacional del Nordeste, Argentina*Correspondence: solebustillo@yahoo.es

The majority of snakebites in northeastern Argentina are caused by *Bothrops alternatus* (yarará grande) and *Bothrops diporus* (yarará chica), reptiles that belong to the Viperidae family. The specific treatment of these ophidian envenomations is serotherapy with antivenoms that ensures a rapid distribution of antibodies and controls systemic alterations but not always the local damage at the bite site where traces of venom are capable of precluding a successful regenerative response. In this work, we explored the characteristics of muscle tissue during the critical period after *Bothrops alternatus* or *Bothrops diporus* venom injection and their potential inhibitory effect on muscle differentiation using an in vitro study model. Groups of CF-1 mice were injected intramuscularly in the right gastrocnemius with 50 µg of B. alternatus or B. diporus venom. Control mice received PBS under identical conditions. Briefly, animals were sacrificed after 0, 1, 3, 24, and 168 h, and muscles were dissected out and placed in liquid nitrogen for pulverization and filtration through 0.22 µm membranes. Venom proteins present in these homogenates were quantified by the ELISA method and analyzed by Western blotting. Myoblast cells (C2C12 cell line) were exposed for 24 h to muscle homogenates, and the less cytotoxic ones were used for myogenesis evaluation. Results evidenced that the amount of both venoms in muscle homogenates decreased over time, with even traces of venom (5–13 µg/mL) being observed 168 h after inoculations. No significant differences were detected between B. alternatus and B. diporus venom treatments. Identification by immunoblotting showed typical venom protein bands with molecular masses between 20 and 100 kDa for B. alternatus and 14 and 100 kDa for B. diporus, whose intensities gradually decreased with time. An intense band of ~60 kDa, characteristic of metalloproteases, was mainly visualized even after 7 days of both treatments. In addition, less cytotoxic muscle homogenates (above 85% of myoblast viability corresponding to 24 and 168 h incubation times) were used for myogenesis assay. Controls showed mature myotube formation after 72 h, but a complete lack of myoblast fusion occurred when myogenic cells were incubated with muscle homogenates from mice injected with bothropic venoms. These preliminary findings suggest that a possible local treatment, complementary to serotherapy, could improve the prognosis of snakebite poisonings by accelerating muscle regeneration processes.

**Keywords:***Bothrops alternatus*; *Bothrops diporus*; myogenesis; muscle regeneration

### 5.12. Exploration of the Biological Effects of a Basic Phospholipase A2 from Agkistrodon Piscivorus Piscivorus Venom

HarveyMerideth^1^*SalazarEmelyn^1^SanchezOscar^1^SuntravatMontamas^1^,^2^SánchezElda E.^1^,^2^*1National Natural Toxins Research Center, Texas A&M University- Kingsville, Kingsville, TX, USA2Department of Chemistry, Texas A&M University- Kingsville, TX, USA*Correspondence: elda.sanchez@tamuk.edu

Background: Phospholipases A2 (PLA2s) are found in abundance in many North American snake species. They are responsible for a wide array of pharmacological effects on tissues both locally and systemically. In *Agkistrodon piscivorus* piscivorus (A.p.p.), these toxins make up a significant portion of venom constituents. A basic PLA2 was recently isolated through reverse-phase HPLC and identified as a D-49 PLA2 (A.p.p PLA2). After testing activities in an in vivo model, the release of pro-inflammatory mediators, systemic myotoxicity, and hemolytic effects were observed. This study aimed to explore the hematological, myotoxic, and pro-inflammatory activities of this toxin using in vitro models. Methods: Whole blood was used to test the hemolytic activity of A.p.p PLA2 in vitro and, through the SONOCLOT analyzer, the effects on the hemostatic system. Moreover, we tested cell viability, expression of cell activation molecules, and cell damage markers on human umbilical vein endothelial cells (HUVECs) representing the vascular system and a myoblast cell line (C2C12) as a muscle model. Cells were incubated with A.p.p PLA2 over different times. Cell viability was tested using MTT assay, and the expression and release of pro-inflammatory and hemostatic mediators were determined using flow cytometry and ELISA. Muscle damage was detected evaluating creatine kinase (CK) release. Discussion/Conclusion: It was observed that A.p.p PLA2 caused significant hemolytic activity and substantial changes in the coagulation system and mild changes in platelet function in whole human blood. Likewise, this toxin altered cell viability in C2C12 cells but not in HUVECs. Endothelial cells were also activated when incubated both at 3 h and at 24 h. Additionally, C2C12 cells released IL-6 and CK, which are markers of cell damage. This data can be used for further experimentation to characterize enzymes belonging to this family to produce specialized antivenoms that target PLA2s and their biological activities.

**Keywords:** phospholipases A2; Agkistrodon piscivorus piscivorus; myotoxicity; hemostatic system; inflammation; cell activation

### 5.13. Biological Activities of Phosphodiesterase from Crotalus durissus Terrificus Venom

FuscoLuciano^1^*HernándezDavid^2^HyslopStephen^2^LeivaLaura^1^*1Laboratorio de Investigación en Proteínas (LabInPro), Universidad Nacional del Nordeste, IQUIBA-NEA, Corrientes Argentina2Departamento de Farmacologia, Faculdade de Ciências Médicas, Universidade Estadual de Campinas (UNICAMP), Rua Tessália Vieira de Camargo, 126, 13083-887, Campinas, SP, Brazil*Correspondence: fuscoluciano@hotmail.com (L.F.); lauraleiva2004@yahoo.com.ar (L.L.)

Phosphodiesterases (PDEs) are an enzyme family that hydrolyze phosphodiester bonds sequentially from the 3’ terminus of polynucleotides to produce 5′-mononucleotides. Historically, snake venom PDEs have been widely used in sequencing and structural studies of nucleic acids. In contrast, the potential pharmacological activities of these enzymes are poorly understood and their role in envenomation remains unclear. Previosuly, we isolated and preliminary characterized a PDE from *Crotalus durissus* terrificus (CDT) venom, demonstrating that is capable of hidrolizing ATP, ADP, AMP, and DNA. Here, we evaluated the edema-forming activity and locomotory behavior induced by CDT-PDE. The enzyme was purified through two chromatographic steps (Sephadex G-75 and HiTrap Q-FF). CDT-PDE activity was tested by chromogenic reaction with sodium salt of bis(p-nitrophenyl phosphate). Groups of five mice were subplantar-injected in the right hind foot with 1 µg of purified PDE or a mixture of PDE (1 μg) and ADP (50 nmol) that was co-injected or adenosine (50 nmol) or ADP (50 nmol) or PBS. Edema was measured as an increase in paw thickness using low-pressure spring calipers at various intervals (0.5, 1, 3, and 6 h). At the end of the experiment, the hind feet were removed and processed for histological analysis. Locomotory behavior was assessed in an open-field test. Each mouse (*n* = 6) received an i.p. injection of PDE or PBS. Mice not injected with PDE or PBS were used as controls. The results indicated that PDE from CDT venom from northeastern Argentina is edematogenic and causes an inflammatory infiltrate. In addition, PDE-CDT reduced the locomotor activity in the initial minutes after injection. All results indicated that PDE exhibits pharmacological activities that should be studied in further detail. Further investigations are required to assess the contribution of this enzyme to the systemic manifestations associated with envenomation by this species.

**Keywords:** snake venom; nucleotidase; nucleotides; edematogenic activity; locomotor activity

### 5.14. Analogous Venom Peptides Acting on Different Pathways: A Study of Bicarinalin and U9-MYRTX-Tb1a from T. bicarinatum Venom

AscoëtSteven^1^*TeneNathan^1^TouchardAxel^1^BarasseValentine^2^LeprinceJérôme^3^BilletArnaud^1^BonnafeElsa^1^TreilhouMichel^1^*1EA-7417, Institut National Universitaire Champollion, Place de Verdun, 81012 Albi, France2Equipe BTSB-EA7417, Université de Toulouse, Institut National Universitaire Jean-François Champollion, Place de Verdun, 81012, Albi, France3InsermU1239, NormandieUniv, UNIROUEN, Plate-forme de Recherche en Imagerie Cellulaire Normandie (PRIMACEN), 76000 Rouen, France*Correspondence: ascoet.pro@gmail.com (S.A.); michel.treilhou@univ-jfc.fr (M.T.)

For decades, venoms have been studied for various applications such as in agriculture, in therapeutics, or as pharmacological tools. Currently, six venom-derived drugs and one venom-derived insecticide are on the market. Ant venoms exhibit a high diversity of peptides, similarly to other arthropod venoms. The first studies have shown many in vitro and in vivo biological effects such as anti-microbial, anti-inflammatory, anti-viral, and even ion channel modulatory effects. Our study focused on M-MYRTX-Tb1a (bicarinalin) and U9-MYRTX-Tb1a, two peptides from Tetramorium bicarinatum venom exhibiting similar sequences. Bicarinalin is an amphipatic α-helical peptide, which was found to forms pores in cell membranes. As the sequence of U9-MYRTX-Tb1a suggests a similar structure with identical physico-chemical properties, we hypothesized a similar biological function. In this study, we investigated the biological effect of U9-MYRTX-Tb1a. We tested the two peptides on Drosophila melanogaster embryonic cells to evaluate their cytotoxicity. As predicted, Drosophila cell lysis was observed with the addition of both peptides. However, the cell morphology after peptide incubation and the time effect was different between U9-MYRTX-Tb1a and bicarinalin, suggesting different mechanisms of action. First, incubation with U9-MYRTX-Tb1a leads to cell growth and bleb formation, with a potential condensation of the nucleus. Then, the cytotoxicity effect of bicarinalin is faster than that of U9-MYRTX-Tb1a. These first results suggest that pro-apoptotic and/or autophagic pathways could be involved in the biological activity of U9-MYRTX-Tb1a. By confirming this hypothesis, we could update the first peptide from ant venom with a pro-apoptotic and/or autophagic effect and the various associated applications.

**Keywords:** ant; venom; peptide; bicarinalin; cytotoxicity; apoptose; autophagy

### 5.15. A Closer Look at the Functional Diversity of Cytotoxins from Southeast Asian Cobras: Biomedical and Clinical Significance

TanChoo Hock^1^*ChongHo Phin^1^TanKae Yi^2^1Venom Research and Toxicology Lab, Department of Pharmacology, Faculty of Medicine, University of Malaya, Kuala Lumpur, Malaysia2Protein and Interactomics Lab, Department of Molecular Medicine, Faculty of Medicine, University of Malaya, Kuala Lumpur, Malaysia*Correspondence: tanch@um.edu.my

The monocled cobra (Naja kaouthia) and the equatorial spitting cobra (Naja sumatrana) are two medically important venomous snakes in Southeast Asia. Proteomics and toxicity studies have established that alpha-neurotoxins are invariably the principal lethal toxins in their venoms. The cytotoxins (cardiotoxins (CTXs)), in contrast, are much complex functionally and structurally. We investigated the venom gene complexity of the cobras through de novo venom gland transcriptomics and showed that cytotoxin genes are the most abundantly and diversely expressed. The CTXs were classified into P-type or S-type CTXs based on the presence of the Pro31 or Ser28 amino acid in the sequence, respectively. To further characterize their pharmacological properties, the CTXs were purified from the venoms through sequential high-performance liquid chromatography, validated with nano-liquid chromatography–tandem mass spectrometry, and investigated for their cytotoxic effects in vitro and in vivo. The P-type and S-type CTXs exhibited differential cytotoxicity, consistent with the variable degree of hydrophobicity in the membrane-binding loop of the toxin molecule. Protein antigenicity was, nevertheless, conserved among the cobra cytotoxins, and this enabled cross-reactivity and cross-neutralization activities of cobra antivenom. The cobra cytotoxins, however, were phylogenetically and immunologically divergent from cytotoxin-like proteins of the Asiatic coral snakes (*Calliophis* spp.), whose cytotoxins form a distinct clade of three-finger toxins with distinct evolutionary implications. Furthermore, the cobra cytotoxins demonstrated high anti-proliferative activities in breast, prostate, and lung cancer cell lines, with promising selectivity noted in the latter. Future studies should aim to unleash the anticancer potentials of the cytotoxins.

**Keywords:** Naja; cardiotoxin; cytotoxin; necrosis; cytotoxic

### 5.16. Engineering of Ribosome-Inactivating Proteins for Improving Anti-HIV Efficacy

LuJia-Qi^1^ZhengYong-Tang^2^ShawPang-Chui^1^*1Centre for Protein Science and Crystallography, School of Life Sciences, The Chinese University of Hong Kong, Shatin, N.T., Hong Kong, China2Kunming Institute of Zoology, Chinese Academy of Sciences, Kunming 650000, China*Correspondence: pcshaw@cuhk.edu.hk

Ribosome-inactivating proteins (RIPs) are N-glycosidases. They depurinate A-4324 in rat 28S ribosomal RNA in the conserved α-sarcin/ricin loop (α-SRL) and cease protein synthesis. Our group has shown that the internal peptide of the maize RIP precursor reduces the anti-HIV activity of the protein in infected macaque peripheral blood mononuclear cells (PBMCs) and the SHIV 89.6-infected Chinese rhesus macaque. We made use of the switch-on mechanism of maize RIP to incorporate HIV-1 protease recognition sequences to its internal inactivation region. Upon activation of this engineered maize RIP by HIV-1 protease in HIV-infected cells, the N-glycosidase activity and inhibitory effect on p24 antigen production in vitro and in infected human T cells were enhanced. This switch-on mechanism could also be applied to the ricin A chain (RTA). RTA variants with the HIV-1 protease recognition sequence at the C-terminus could be cleaved both in vitro and in HIV-infected cells. Furthermore, its antiviral effect was enhanced, and the cytotoxicity toward uninfected cells was reduced. Our study provides a platform technology in creating protein toxin derivatives with increased pathogen-specific cytotoxicity.

**Keywords:** ribosome-inactivating protein; anti-HIV; engineering; switch-on mechanism

## 6. Use of Toxins as Tools for Research, Drug Discovery, and Therapeutics

### 6.1. Engineering the NADPH Specificity of DepB, a Novel Aldo-Keto Reductase Involved in the Detoxification of the Agroeconomic Mycotoxin Deoxynivalenol (DON)

AbrahamNadine^1^,^2^CarereJason^2^SeahStephen Y. K.^1^*ZhouTing^2^*1Department of Molecular and Cellular Biology, University of Guelph, Guelph, ON, Canada2Guelph Research and Development Centre, Agriculture and Agri-Food Canada, Guelph, ON, Canada*Correspondence: sseah@uoguelph.ca (S.Y.K.S.); ting.zhou@canada.ca (T.Z.)

Deoxynivalenol (DON) is a toxic fungal secondary metabolite produced by Fusarium graminearum, which causes Fusarium head blight and pink ear rot diseases in wheat and corn, respectively. DON is a predominant contaminant in cereal grain crops, with outbreaks costing the North American cereal grain industry millions of dollars annually. There is a growing need for effective DON mitigation strategies due to DON’s inherent toxicity, which affects the performance of livestock fed contaminated grain. Current DON management strategies involve physical decontamination and marginally effective chemical treatments; however, a holistic and targeted approach via the incorporation of DON-detoxifying enzymes is a promising strategy. Previous studies have demonstrated that D. mutans 17-2-E-8, a soil bacterium, epimerizes DON to the less toxic 3-epi-DON via the intermediate, 3-keto-DON. The process involves two enzymes, DepA, a PQQ-dependent dehydrogenase, and DepB, an NADPH-dependent aldo-keto reductase (AKR). The strict requirement for the expensive cofactor, NADPH, poses a significant impediment to the practical application of these enzymes. Protein engineering approaches can address this issue: by switching DepB’s cofactor preference to the cheaper cofactor, NADH. DepB was found to catalyze the transformation of 3-keto DON to 3-epi DON with Km and kcat values of 563.9 µM and 2.49 s^−1^, respectively, using NADPH as a cofactor. Second, the enzyme’s Kd for NADPH was determined to be 44.23 µM using fluorescence enhancement assays. Using the solved crystal structure of DepB, docking experiments with DepB revealed that Arg-289, Gln-293, and Lys-216 may be important for NADPH specificity. Therefore, site-specific mutagenesis was performed to replace these residues to enable the enzyme to use NADH. The catalytic efficiencies for these designed mutants will next be determined and compared to catalytic efficiencies of the wild-type DepB.

**Keywords:** deoxynivalenol (DON); 3-keto-DON; 3-epi-DON; detoxification; mycotoxin; aldo-keto reductase

### 6.2. Potential of Cyanobacterial Extracts for Anticancer Activity

PorzaniSamaneh JafariNowruziBahareh*Department of Biology, Science and Research Branch, Islamic Azad University, Tehran, Iran*Correspondence: bahare77biol@yahoo.com

Natural bioactive compounds derived from living organisms have traditionally been used as medicine. Being factories for several bioactive compounds, cyanobacteria may be a potential target to treat several diseases, including cancers. Several studies have been undertaken during the past three decades to find the anticancer property of cyanobacterial toxins. These have led to the discovery of several promising molecules having anticancer activity, some of which are in clinical trials and may emerge to be future drugs in cancer therapy. These compounds are useful tools against cancerous cell lines as a promising medicine. For example, cryptophycins from *Nostoc* sp. have been explored as a promising anticancer agent for lung cancer and in patients with platinum-resistant advanced ovarian cancer. Borophycin from *Nostoc linckia* and *Nostoc spongiforme* sp. demonstrates effective bioactivity toward human epidermoid carcinoma (LoVo) and human colorectal adenocarcinoma (KB) cell lines, along with the antimicrobial activity. Apratoxin from the marine cyanobacterium Lyngbya majuscula was also effective against LoVo and KB cancer cell lines. The methylene chloride/methanol-soluble extract of cyanobacteria, *S. hofmanni* species, named nostodione was also found to inhibit the chymotrypsin-like activity of the proteasome in vitro. Cyanotoxins provide a great proportion of new therapeutic drugs. Hence, an understanding of natural toxins may help to facilitate the combating of detrimental effects of conventional cancer therapy in the future.

**Keywords:** natural compound; toxins; cyanotoxins; anticancer activity

### 6.3. Role of Cyanobacterial Toxins as a Source against Coronavirus Disease Treatment

PorzaniSamaneh JafariNowruziBahareh*Department of Biology, Science and Research Branch, Islamic Azad University, Tehran, Iran*Correspondence: bahare77biol@yahoo.com

In recent years, cyanobacterial bioactive compounds have drawn research interest for two main reasons: severe toxicity of toxins released by many freshwater blooming cyanobacteria and their adverse effects on animals and human health, and potential therapeutic use of certain secondary metabolites. Secondary cyanobacterial metabolites include a number of compounds that demonstrate animal toxicity and antibacterial, anticoagulant, antifungal, anti-inflammatory, antimalarial, antiprotozoal, antituberculosis, antiviral, and antitumor activities. There has been a surge in antiviral compounds from natural resources, along with some therapies. In this context, cyanobacterial antiviral compounds have emerged as primary compounds for antiviral treatment. Cyanobacteria belonging to the genera Calothrix, Microcystis, Nodularia, Nostoc, Spirulina, Oscillatoria, and Scytonema have been identified as sources of antiviral compounds. Natural toxins from cyanobacterial species, especially cyanotoxins, have shown activity against severe acute respiratory syndrome (SARS) virus. For example, cyanobacterial species in Chroococcales, Pleurocapsales, Oscillatoriales, Nostocales, and Stigonematales show activity against two SARS viral proteases: the papain-like protease and the chymotrypsin-like protease. Cyanobacterial extracts could have a potential effect on inhibiting these viral proteases from the coronavirus (CoV) responsible for severe acute respiratory syndrome (SARS). There is promising inhibitory ability against SARS-CoV-2 shown by the metabolites cylindrospermopsin, deoxycylindrospermopsin, carrageenan, cryptophycin 52, eucapsitrione, tjipanazole, tolyporprophin, and apratoxin A. Cryptophycin 1, cryptophycin 52, and deoxycylindrospermopsin compounds demonstrate encouraging binding energy scores with the SARS-CoV-2 PLpro. The most successful nominee antagonist against both SARS-CoV-2 proteases is deoxycylindrospermopsin. The findings provide ample space for exploiting the ability of deoxycylindrospermopsin as an effective in vitro and in vivo SARS-CoV-2 inhibitor and pave the way for the production of novel, powerful COVID-19 therapeutics. Cyanobacterial antiviral compounds have been among natural compounds as antiviral tools. The research on the use of these compounds in fighting a number of viruses has laid the foundation for the development and use of antiviral compounds from cyanobacteria for SARS-CoV-2-induced COVID-19.

**Keywords:** cyanotoxins; natural compounds; coronavirus; SARS-CoV-2

### 6.4. Free-Binding Energies and Molecular Interactions of Yessotoxin in the Voltage-Gated Sodium Channel NaV1.5: An In Silico Approach

Osorio-RamírezM. Carmen^1^Durán-RiverollLorena M.^2^,^3^*CembellaAllan D.^3^Correa-BasurtoJosé^1^1Laboratorio de Diseño y Desarrollo de Nuevos Fármacos e Innovación Biotecnológica, Sección de Estudios de Posgrado e Investigación, Escuela Superior de Medicina, Instituto Politécnico Nacional, Ciudad de México, Mexico2CONACyT-Departamento de Biotecnología Marina, Centro de Investigación Científica y de Educación Superior de Ensenada, B.C. Ensenada, Baja California, México3Alfred-Wegener-Institut, Helmholtz-Zentrum für Polar-und Meeresforschung, Bremerhaven, Germany*Correspondence: lduran@conacyt.mx

Several marine dinoflagellates produce unique secondary metabolites with intriguing biological activities, eliciting anticancer, anti-epileptic, anti-inflammatory, or anti-microbial responses in various cell types. Among these known compounds are phycotoxins, such as linear and cyclic polyethers considered potential therapeutants due to their complex alternative mode of action as ion-channel effectors or enzyme inhibitors capable of modifying diverse intracellular signaling pathways. Yessotoxin (YTX) and analogues are polyketide-derived polycyclic toxins produced by certain species of marine dinoflagellates, Protoceratium reticulatum, Lingulodinium polyedra, and Gonyaulax spinifera, and are structurally related to ciguatoxins and brevetoxins with potent ion-channel activity. The over-expression or aberrant function of ion channels is considered a channelopathy, and critical pathologies in different intracellular signaling pathways that involve receptors such as voltage-gated ion channels, particularly voltage-gated sodium channels (NaV), are exhibited in response to exposure to such toxins. In the search for innovative therapeutants, this study aimed to evaluate the affinity of YTX for the NaV1.5 channel using in silico modeling tools. This approach allowed for identification of these interactions and determination of the respective free-binding energies. Our results showed significant interactions and low free-binding energies (ΔG), between −6.79 and −10.32 Kcal mol^−1^ for YTX in the NaV1.5 protein model. Certain amino acid residues in domains I and II were reached, indicating that this toxin is a potential NaV 1.5 modulator. This study constitutes the first approach to in silico exploration of polyketide-derived dinoflagellate toxins in pursuit of evaluating their therapeutic potential.

**Keywords:** biological activity; free-binding energy; ion channel modulator; molecular docking

### 6.5. Bothrops moojeni Venom: A New Tool to Investigate Osteoclast Differentiation

D’AmélioFernanda^1^,^2^*BarrosHugo Vigerelli de^1^,^3^SilvaÁlvaro Rossan de Brandão Prieto da^1^Fra-reEduardo Osório^1^,^3^BatistaIsabel de Fátima Correia^3^PimentaDaniel Carvalho^4^KerkisIrina^1^,^3^1Laboratory of Genetics, Butantan Institute, São Paulo, Brazil; Laboratory of Biochemistry, Butantan Institute, São Paulo, SP, Brazil2The Postgraduate Program in Toxinology, Butantan Institute, São Paulo, Brazil3Centre of Excellence in New Target Discovery (CENTD), Butantan Institute, São Paulo, Brazil4Laboratory of Biochemistry, Butantan Institute, São Paulo, Brazil*Correspondence: fernanda.damelio@butantan.gov.br

*Bothrops moojeni*, a Brazilian lanced-head viper, presents a rich, but not well explored, venom composition. This venom is a powerful tool for the discovery of new molecular targets in many different biological processes. Osteoclasts (OCs) are extremely important for bone maintenance, calcium physiology, and balance of tissue regeneration, being involved in such diseases as osteoporosis and rheumatoid arthritis. The goal of our study was to evaluate the effect of *Bothrops moojeni’s* venom and its fractions on human peripheral blood mononuclear cell-derived OCs’ in vitro differentiation. After the induction of OC differentiation, on day 4, the venom was added at different concentrations (5, 0.5, and 0.05 µg/mL), and a reduction in tartrate-resistant acid phosphatase-positive (TRAP+) osteoclasts, which was more prominent at the concentration of 5 µg/mL, was observed. Phalloidin staining was used for morphological analyses of F-actin ring integrity. The venom provoked F-actin ring disruption in treated versus control OCs. We obtain high-molecular-weight (HW) and low-molecular-weight (LW) venom fractions. Both fractions induced a reduction in TRAP+ OCs (HW fraction at a concentration of 5 µg/mL and LW fraction at 1 µg/mL). We performed secretome analysis of OCs treated with venom and its fractions using mass spectrometry (LC-MS/IT-Tof). The data obtained demonstrate possible pathways and mechanisms involved in OCs’ reduction after treatment, for example, catabolic mechanisms for HW venom fractions and proteins correlated with genetic modifications for LW venom fractions. New experiments are in progress, aiming to discover molecules that possibly interfere with osteoclast differentiation.

**Keywords:** Bothrops moojeni; osteoclasts; cell differentiation

### 6.6. Production of Monoclonal Antibody (mAb)-Purified Anti-Metalloprotease from the Venom of the Serpent Bitis arietans

GodoiKemily Stephanie de^1^*GuidolinFelipe Raimondi^1^MegaleÂngela Alice Amadeu^2^PortaroFernanda Vieira Calheta^3^SilvaWilmar Dias da^1^*1Development and Innovation Division, Immunochemistry Laboratory—Butantan Institute2Biological Quality Control—Butantan Institute3Development and Innovation Center—Blood Products Laboratory—Butantan Institute*Correspondence: kemily.godoi@butantan.gov.br (K.S.d.G); wilmar.silva@butantan.gov.br (W.D.d.S.)

The African snake *Bitis arietans* is of great medical importance and is found in sub-Saharan Africa and in savannas and pastures of Morocco and western Arabia. It contributes significantly to the epidemiology of snakebites in humans and animals. The lack of specific antivenoms aggravates this situation. Identifying toxins, knowing their toxic properties, and developing antitoxins are the goals of emerging projects. This study aims to extract monoclonal antibody (mAb)-purified anti-metalloprotease from the toxin of *Bitis arietans* venom. mAbs serve as sources of complementarity-determining regions (CDRs). The main applied methodologies are mainly for the purification of metalloprotease and the immunization of mice to obtain lymphocytes and replicate them. So far, we have been able to highlight a metalloprotease of interest, which will be properly identified for the production of antibodies, a finding that was confirmed by proteomic and transcriptomic analyses. The next step will be to immunize mice and validate the antibodies produced.

**Keywords:***Bitis arietans*; metalloprotease; monoclonal antibodies; complementarity-determining regions

**Key Contribution:** The main contribution of the study so far has been the finding of a purified SVMP and the F5-4 fraction. From this, we can also find an SVSP, take advantage of other emerging projects, as well as standardize our purification protocols. These findings will contribute to the next experimental steps.

### 6.7. Synergistic Attenuation of Cancer-Related Pain and Implications for Adverse Effects of the Use of Methadone and Phα1β in C57BL/6J Mice

AssisLuana*GomezMarcus ViniciusJuniorCélio de CastroPrograma de pós graduação em ciências da saúde, Santa Casa de Belo Horizonte Ensino e Pesquisa, Belo Horizonte, Minas Gerais, Brazil*Correspondence: luana.assisferreira@gmail.com

Introduction and Goals: Cancer pain produces severe distress and lowers the life quality of patients and often is not effectively treated. Opioids are practically the only analgesics capable of controlling cancer pain, but this therapy leads to distinct side effects that limit opioid use. Methadone is a valuable opioid analgesic, which can be administered in the case of cancer pain and can reverse tolerance to other opioids like morphine. However, methadone has some side effects like other opioids. Phα1β toxin from the spider *Phoneutria nigriventer* has an antinociceptive action in several models of pain in rodents, and it is known that it induces analgesic effects in a model of cancer pain in mice. This toxin is a dual blocker of TRPA1 channels and voltage-gated calcium channels and exhibits greater selectivity for N-type channels. One strategy to improve the therapeutic utility of opioids is to co-administer them with other analgesic agents, such as Phα1β toxin, looking for overall dose reduction and also reducing side effects to improve the quality of analgesia. This work aims to analyze by isobolographic analysis whether antinociceptive interaction of methadone and Phα1β is sub-additive, additive, or synergic. Methodology: B16F10 cells were inoculatedinto the right paw on C57BL/6J mice for tumor induction. The PWT (von Frey filaments) was measured before (baseline), at day 7, and at day 14 before and after drug treatment (N = 6–9 per group). Dose–response curves of drugs alone or in combination were performed using a fixed proportion design. Data interpretation was performed using isobolographic analysis to determine the interaction index of the combination. To evaluate the possible side effects of this combination, the open field test, the rotarod test, and quantification of gastrointestinal transit were performed. To check whether the combination is capable of reversing morphine tolerance induced by several morphine doses, the protocol with tail-flick apparatus was used. All the procedures were authorized by CEPEEA, the ethics committee for animals’ experimentation from Santa Casa of Belo Horizonte Education and Research (Protocol 002/2018). Results: Fourteen days after right hind paw inoculation with B16F10 cells, marked hyperalgesia was induced, as measured by von Frey filaments. This hyperalgesia was reversed by i.t. treatment with Phα1β at 100 pmol/site and also by methadone s.c. injection at 1 mg/kg; other doses were tested, and the final dose used to combine these two drugs was decided, as described by Tallarida in a protocol of fixed proportions by two components. The antinociceptive effect of Phα1β and methadone was dose dependent, with ED50 values of 1.076 pmol/site for Phα1β and 86.849 pmol/site for methadone. The combination of Phα1β and methadone had an ED50 lower than the theoretical additive ED50 (*p* < 0.05), indicating synergism. The required dose to reach synergism was 20 pmol/site for Phα1β (95% CI: 7–58 pmol/site) and 15.410 pmol/site for methadone (95% CI: 5.480–41.680 pmol/site). Since we observed strong potentiation of the analgesic effect, we assessed the animal behavior in an open field, as well as motor impairment, checking fall latency and intestinal motility after the administration of drugs alone or in combination at ED50 doses. No changes in animal behavior were observed in an open field (traveled distance, movement number, and movement time duration) after drug administration (*p * > 0.05); the same occurred in the latency to fall: no difference was seen between groups (*p * > 0.05). Combined drugs reduced 27% of gastrointestinal transit compared with the control group (*p* = 0.02), whereas the two drugs alone did not significantly differ from the control group (*p* < 0.05). Methadone is currently used to reverse morphine tolerance, and methadone given alone at its ED50 dose was able to reverse morphine-induced tolerance in animals, with a significant difference (*p* < 0.05) from the control group (PBS). Phα1β also reversed morphine tolerance, as described earlier. At lower doses, the Phα1β + methadone ED50 value reversed morphine tolerance, without a significant difference (*p* > 0.05) from isolated compounds but with a statistical difference from the control group (*p* < 0.05). Conclusions: Our data show that synergism occurs when s.c. methadone is administered simultaneously with i.t. Phα1β, suggesting potentiation of the analgesic effect of these drugs when added together. Even with strong potentiation of the analgesic effect, no relevant side effects associated with this combination sre observed. In addition to producing an antinociceptive effect, the combination of these compounds is able to reverse morphine-induced tolerance.

**Keywords:** cancer pain; melanoma; Phα1β toxin; methadone; synergism; isobolographic analysis

### 6.8. The Antitumoral Potential of Pllans-II, an Acidic PLA2 from Porthidium lansbergii Lansbergii Snake Venom, in Human Cervical Carcinoma Cells

Montoya-GomezGabriel Alejandro^1^*Montealegre-SanchezLeonel Ives^1^Castillo-GiraldoAndres Orlando^2^Rivera-FrancoNelson^3^CharrisEliécer Jiménez^1^*1Grupo de Nutrición, Facultad de Salud, Universidad del Valle, Cali, Colombia2Departamento de Biología, Facultad de Ciencias Naturales y Exactas, Universidad del Valle, Cali, Colombia3Laboratory of Human Molecular Genetics, Department of Biology, Universidad del Valle, Cali, Colombia*Correspondence: gabriel.montoya@correounivalle.edu.co (G.A.M.-G.); eliecer.jimenez@correounivalle.edu.co (E.J.C.)

Pllans-II, an Asp49-type acidic phospholipase A2 from *Porthidium lansbergii* lansbergii snake venom, displayed for the first time antitumoral potential against the cervical cancer Ca Ski squamous epithelial cell line. Pllans-II presented a dose-dependent cytotoxic effect on cancer cells and an insignificant effect on healthy human umbilical vein endothelial cells (HUVECs). Pllans-II also inhibited the adhesion and migration ability of cancer cells and induced cell cycle arrest in the G2/M phase and apoptosis of Ca Ski cells. Transcriptomic analysis revealed that cell death was related to endoplasmic reticulum stress by interfering with α5- and β1-containing integrins. These results demonstrate that Pllans-II has antitumor potential in cervical cancer and represents a possible biotechnological tool for designing anticancer prototypes.

**Keywords:** cervical cancer; cytotoxicity; apoptosis; endoplasmic reticulum stress; phospholipase A2

### 6.9. Linear Scorpion Peptides: An Unexplored Pool for Peptide Hydrogels

AvraamidesConstantinos^1^DiavoliSpiridoula^1^RobertsonAriana^2^VlasiouManos^1^MourelatouElena^1^PetrouChristos^1^SarigiannisYiannis^1^*1Department of Life & Health Sciences, University of Nicosia, Nicosia, Cyprus2Department of Molecular and Cell Biology, University of California Berkeley, USA*Correspondence: sarigiannis.i@unic.ac.cy

Scorpions, during their long evolutionary existence on the planet, more than 400 million years, have managed to develop a series of venom peptides that display diverse biological activities and pharmacological functions. Scorpion venom peptides are generally classified into two main groups: disulfide bridged peptides (DBPs), which usually target membrane-bound ion channels, and non-disulfide bridged peptides (NDBPs), a smaller group with multifunctional activities. Our current study focuses on short (13–19 amino acids) antimicrobial linear scorpion peptides. Many of these peptides contain sections, ranging from short to long, of identical amino acid sequences. Most of them display a net positive charge of 1 or 2, exhibit an isoelectric point at pH 9–10, have a broad range of hydrophobicity, and a grand average of hydropathy (GRAVY). These features allow these peptides to be attracted toward the negatively charged phospholipid head groups of the lipid membranes of target cells, a force driven by electrostatic interactions. Here, we present the synthesis of mucroporin peptide, a 17-amino-acid linear peptide isolated from the venom of Lychas mucronatus, and its synthetic analogues. Mucroporin exhibited a positive charge of 1 due to a lysine at the C-terminus site of the peptide. The rest of the peptides were mainly aliphatic leucine, glycine, or isoleucine. A series of synthetic analogues were designed, synthesized, purified, and characterized by liquid chromatography—mass spectrometry (LC-PDA-MS) and nuclear magnetic resonance (NMR) spectroscopy. Mucroporin as well as its synthetic analogues were tested with various techniques for their ability to form hydrogels under several conditions.

**Keywords:** mucroporin; non-disulfide bridged peptides; peptide hydrogels; scorpion peptides; dynamic light scattering

### 6.10. Shedding New Light on the Recombinant β-KTx Neurotoxin from Tityus serrulatus: Heterologous Expression and Structural and Functional Characterization

AmorimFernanda Gobbi^1^*FrançaJohara Boldrini^2^CordeiroFrancielle Almeida^3^CerniFelipe Augusto^3^PuccaManuela Berto^4^PeigneurSteve^5^TytgatJan^5^EchterbilleJulien^1^QuintonLoïc^1^ArantesEliane Candiani^3^1Laboratory of Mass Spectrometry, Department of Chemistry, University of Liège, Liège, Belgium2Postgraduate Program in Ecosystem Ecology, Vila Velha University, Vila Velha, Brazil3School of Pharmaceutical Sciences of Ribeirão Preto, University of São Paulo, Ribeirão Preto, Brazil4Medical School, Federal University of Roraima, Boa Vista, Brazil5Toxicology and Pharmacology, University of Leuven, Leuven, Belgium*Correspondence: fernandagamorim@gmail.com

Neurotoxins are majorly responsible for the symptoms caused by *Tityus serrulatus* envenoming due to their actions on ion channels of excitable cells. However, structural and functional analyses of these toxins are difficult due to the low amount of purified toxin obtained from the crude venom. The combination of -omics techniques allows the precise identification of novel components with biotechnological applications, enabling their heterologous expression. We reported the heterologous expression of recombinant Ts19 (rTs19), a β-KTx neurotoxin, and its structural and functional characterization. The cDNA encoding rTs19 was obtained from the *Tityus serrulatus* venom gland transcriptome, cloned into the pPICZαA plasmid, and transformed into cells of the KM71H *Pichia pastoris* strain. rTs19 was purified by immobilized metal affinity and C18 chromatography procedures and showed higher expression after 96 h of induction in buffered methanol-complex medium at 30 °C. The expression of the toxin was confirmed by Western blot using anti-His-tag antibody. In addition, rTs19 showed a molar mass of 6555.05 Da confirmed by FT-ICR high-resolution mass spectrometry (Solarix, Bruker). After reduction and alkylation, MALDI-TOF analyses (Ultraflex II, Bruker) confirmed the three disulfide bridges of the toxin. rTs19 was sequenced by enzymatic digestion using trypsin and MS/MS fragmentation in a Q-TOF mass spectrometer (SynaptG2, Waters). Electrophysiological experiments and a voltage clamp with two microelectrodes on Xenopus laevis oocytes were performed to screen the action of rTs19 over 16 different subtypes of Kv channels. rTs19 interacted with potassium channels, blocking Kv1.4 and hERG channels with high potency. These results demonstrated the first recombinant expression of a β-KTx neurotoxin from *Tityus serrulatus*. The *P. pastoris* expression system seems to be an efficient, rapid, and cheap method for obtaining such toxins using a recombinant methodology. Furthermore, these results may open new perspectives of bioprospection of the biological actions of rTs19.

**Keywords:***Tityus serrulatus* venom; heterologous expression; neurotoxin

### 6.11. Expression and Purification of rTs7, a Recombinant Toxin from Tityus serrulatus Scorpion Venom

JacobBeatriz de Cássia da SilvaCordeiroFrancielle AlmeidaCardosoIara AimêWiezelGisele AdrianoArantesne Candiani*Department of BioMolecular Sciences, School of Pharmaceutical Sciences of Ribeirão Preto, University of São Paulo, Ribeirão Preto, SP, Brazil*Correspondence: ecabraga@fcfrp.usp.br

*Tityus serrulatus* venom is composed of several substances, including neurotoxins that interact with voltage-gated ion channels. These channels are involved in many diseases, such as arrhythmia, asthma, autoimmune diseases, hypertension, and immune response to infection and inflammation, making T. serrulatus venom an important tool to study them. Ts7, also called TsTx-K-alpha, acts selectively on potassium channels and can contribute to the treatment of Kv1.3 channel-related diseases, this channel being a potential therapeutic target in the treatment of autoimmune diseases. In this work, we present the heterologous expression of Ts7 in *Pichia pastoris* yeast and its purification. The toxin gene was synthesized with the tobacco etch virus (TEV) protease cleavage site before the N-terminal sequence and cloned into the pPICZαA vector. *P. pastoris* cells (KM71H strain) were transformed with the linearized plasmid rTs7_pPICZαA by electroporation. Transformation was confirmed by PCR of selected colonies and 1% agarose gel electrophoresis. Positively transformed colonies were submitted to a screening in a 24-well plate under standard conditions (pH 6 for 144 h) in order to determine the maximum expression rate. The colony showing the highest expression level of the recombinant protein was selected for laboratorial-scale expression, and the progress of expression was monitored by SDS-PAGE. The expressed protein was purified through immobilized metal affinity chromatography (IMAC) followed by reverse-phase chromatography on a C-18 column. In the reverse-phase chromatography, three fractions were observed, and after mass spectrometry analysis, rTs7 was identified in fraction 3, and fractions 1 and 2 were possibly the cleaved toxin. rTs7 was successfully expressed and purified, with a satisfactory yield of the recombinant toxin, which showed high similarity with the native toxin. The rTs7 immunosuppressive activity in a multiple sclerosis model will be further investigated.

**Keywords:***Tityus serrulatus*; heterologous expression; *Pichia pastoris*; recombinant toxin

**Financial support:** CAPES, CNPq, FAPESP.

### 6.12. Heterologous Expression of a Neurotoxin from Tityus serrulatus Scorpion Venom in Pichia pastoris Yeast and the Evaluation of Its Glycosylation Patterns

CordeiroFrancielle Almeida^1^*AmorimFernanda Gobbi^2^Boldrini-FrançaJohara^3^Pinheiro-JuniorErnesto Lopes^4^CardosoIara Aimê^1^PeigneurSteve^4^TytgatJan^4^ArantesEliane Candiani^1^1Department of BioMolecular Sciences, School of Pharmaceutical Sciences of Ribeirão Preto, University of São Paulo, Ribeirão Preto, SP, Brazil2Laboratory of Mass Spectrometry, Department of Chemistry, University of Liège, Liège, Belgium3Postgraduate Program in Ecosystem Ecology, Vila Velha University, Vila Velha, Brazil4Toxicology and Pharmacology, University of Leuven, Leuven, Belgium*Correspondence: fran_acordeiro@hotmail.com

*Tityus serrulatus* is the most dangerous species of scorpion in Brazil. Its venom (TsV) has mainly neurotoxins, which can act on sodium or potassium channels and are responsible for most envenoming symptoms. The evaluation of these toxins can elucidate their mechanisms as well as contribute to a more specific therapy. The aim of this study was the expression of Ts15, an α-KTx from TsV, in *Pichia pastoris* and its characterization. The rTs15 gene was synthetized by GenScript^®^ with the tobacco etch virus (TEV) protease cleavage site before the N-terminal sequence and cloned into the pPICZαA vector. The recombinant plasmid was transformed in the KM71H Pichia strain, and the screening of positive colonies was performed in a deep-well plate. The laboratory-scale expression was first performed in glycerol medium and methanol medium for induction. Peptide expression was analysed by SDS-PAGE (16%) with silver stain and Schiff reagent that specifically stain carbohydrates. We also performed spectrometry analysis of toxins in MALDI-TOF equipment, a N-glycosylation reaction with PNGase enzyme, and electrophysiological analysis in Kv 1.1, 1.2, 1.3, and 2.1 using the two-microelectrode voltage clamp technique. SDS-PAGE revealed three bands, and their molecular masses by spectrometry analysis were 7.76, 7.5, and 5.5 kDa. The Schiff stain revealed that the toxins with 7.76 and 7.5 kDa were glycosylated, and the reaction with PNGase was able to remove part of this glycosylation, indicating that *P. pastoris* performs N-glycosylation. A preliminary electrophysiological screening with non-glycosylated toxin showed low inhibition in Kv 1.3. In conclusion, rTs15 was successfully expressed in *P. pastoris* yeast, as well as two glycosylated forms of the toxin, and the low inhibition in Kv 1.3 is probably due to the recombinant N-terminal. As the next steps, the same tests will be performed with glycosylated and cleaved toxins.

**Keywords:** Tityus serrulatus venom; α-KTx; Ts15; heterologous expression; glycosylation

### 6.13. Use of Neostigmine–Atropine Plus Antivenom in the Experimental Envenomation by Micrurus Venom. Preliminary Results

RoodtAdolfo de^1^,^2^*DozoretzDaniel^2^GoñiFernando Morón^2^DesioMarcela Alejandra^1^LanariLaura Cecilia^1^DaminCarlos Fabián^2^1Instituto Nacional de Producción de Biológicos ANLIS “Dr. Carlos G. Malbrán”. Ministerio de Salud, Argentina2Facultad de Medicina, Universidad de Buenos Aires*Correspondence: aderoodt@gmail.com

Venoms of most elapids are neurotoxic, their most important components being alpha-neurotoxins and phospholipases A2 (PLA2s). Treatment with neostigmine and atropine (NA) has been suggested to revert the toxicity of nicotinic toxins. The usefulness of an alternative tool is important due to the lack of antivenom for some elapids like Micrurus (M.) due the scarcity of specific antivenoms (AV). We assayed in rescue experiments (mice challenged with mortal doses) the usefulness of the combination neostigmine–atropine (NA) alone or combined with AV, the venoms of *Naja (N.) kaouthia*, *M. altirostris*, *M. pyrrhocryptus*, and *M. surinamensis*. The antivenoms used were therapeutic anti-Micrurus and experimental anti-Naja siamensis antivenoms. Despite that, all the cases received a single dose of 20 μg of atropine + 2.5 μg of neostigmine by the i.p. route, which delayed the time of death (*p* < 0.05), but no good protection was observed using only this treatment. In contrast, only high doses of AV achieved some level of protection. Nevertheless, the combination of NA plus AV reduced mortality, as well as the dose of antivenom required for protection in all the cases regarding these treatments used alone. In the case of *M. altirostris* venom, the protection using NA was from 0% to 20% and that using 50 μL of AV ranged from 0% to 60%; while using the combined treatment, the protection was from 80% to 100% (*p* = 0.046 and 0.02 regarding AV or NA alone). In other cases, an improvement was observed with the use of NA alone, AV alone (250 μL), or their combination. In the case of *N. kaoutia*, the protection was 0%, 20%, and 40%, respectively; *M. pyrrhocryptus*, 0%, 60%, and 100%, respectively; and *M. surinamensis*, 0%, 0% to 20%, and 40%–80%, respectively. These preliminary results suggest the utility of this combination for the treatment of these envenomations, which could be helpful to reduce the dose of AV.

**Keywords:** elapids; Micrurus; neostigmine; atropine; envenomation; antivenom; treatment

### 6.14. Paraspecific Neutralization of the Venom from Adults and Young Crotalus atrox by Paraspecific South American Antivenoms

RoodtAdolfo de^1^,^2^*DesioMarcela Alejandra^3^LanariLaura Cecilia^3^LagoNéstor Rubén^4^GoñiFernando Morón^5^DozoretzDaniel^5^CalderónLeandro^3^RegnerPablo^5^OliveiraVanessa Costa de^5^DaminCarlos Fabián^5^1Ministry of Health, University of Buenos Aires2Laboratorio de Toxinopatología, Centro de Patología Experimental y Aplicada, Facultad de Medicina, Universidad de Buenos Aires3Área Investigación y Desarrollo—Venenos, Instituto Nacional de Producción de Biológicos, ANLIS “Dr. Carlos G. Malbrán”, Ministerio de Salud y Desarrollo Social, Argentina4Centro de Patología Experimental y Aplicada, Facultad de Medicina, Universidad de Buenos Aires, Argentina5Primera Cátedra de Toxicología, Facultad de Medicina, Universidad de Buenos Aires, Argentina*Correspondence: aderoodt@gmail.com

*Crotalus atrox* is one of the species of venomous snakes most commonly found in herpetological collections around the world, and it is usually commercialized in the black market. Several collections have specimens but lack specific antivenoms. We tested the toxicity of the venoms of adult and young (2 to 3 years old) specimens of C. atrox in captivity and the para-specific neutralization provided by the antivenoms most used in Argentina. The i.p. lethal potency of the venoms were 100(95–105) μg and 43(42–45) μg per 20 g mouse, and the indirect hemolytic activity was 7.9 (6.7–9.2) μg and 9.0(8.3–9.9) μg for adult and juvenile venoms. Despite the adult venom’s lower lethal potency, it was more difficult to neutralize: around 1.5 mL of antibothropic (AB) antivenom was necessary to neutralize 1 mg of venom in contrast to 0.54 mL required to neutralize young specimens’ venom. Neutralization by the anticrotalic (AC) antivenom was ineffective. The dose of AB required for neutralization of 5-fold LD50 venom of young snakes was in the range of that required for the neutralization of specific venoms; nevertheless, the dose required to neutralize venom from adults was 6-fold higher. The experiments using 2-fold LD50 as the challenge dose showed similar results. The indirect hemolysis caused by both venoms was similarly neutralized by AB (*p* < 0.05), while AC did not show neutralizing activity. The myotoxicity determined by the increase in creatinquinase or by histopathology was neutralized by both antivenoms, possibly due to the presence of myotoxins like K49 phospholipases present in the venoms. Although the paraspecificity of AB has a potential use as treatment, especially in the case of young snakebites, the doses required in adult attacks are high. Although AB seems useful for emergencies, these results suggest advantages of using a specific antivenom for the treatment of these snakebites.

**Keywords:***Crotalus atrox*; venom; antivenom; antibothropic; anticrotalic; toxicity; treatment

## 7. Impact of Toxins on Public Health

### 7.1. Herbal Tea: Transfer of Mycotoxins from Matrix into Infusion

KiselevaMariya*ChalyyZakharSedovaIrinaFederal Research Centre of Nutrition and Biotechnology, Moscow, Russian Federation*Correspondence: mg_kiseleva@mail.ru

Herbal supplements are natural products that are traditionally considered helpful or at least harmless for health promotion. Consumption of herbal products has increased, but their safety, especially mycotoxin contamination, is still poorly controlled. Recent surveys report the occurrence of *Aspergillus* and *Penicillium* metabolites (aflatoxins (AFLs), ochratoxin A (OTA), cyclopiazonic and mycophenolic acids (MPA), sterigmatocystin (STE), citrinin), *Fusarium* (trichothecenes, zearalenone (ZEA), fumonisins (FBs), enniatins (ENNs)), and *Alternaria* (alternariol (AOH), its methyl ether (AME), tentoxin (TTX), and tenuazonic acid) toxins. A significant part of herbal supplements is consumed in the form of infusions. Thus, correct risk assessment needs evaluation of mycotoxin transfer rates from the herbal matrix into the solution. We studied the transfer of AFLs, OTA, STE, deoxynivalenol (DON), ZEA, FBs, T-2 and HT-2 toxins, AOH, AME, TE, ENNs, beauvericin, and MPA from the spiked herbal matrix into the infusion at different pH values and the total dissolved solids (TDS) characteristics of the water used for its preparation. Analytes were detected by HPLC-MS/MS. The transfer rate proved to be dependent on the mycotoxins’ polarity and pH of the resulting infusion. The TDS did not affect transfer significantly. ENNs, BEA, STC, ZEA, and AOH transfer into the infusion was below 25%; AFLs, 25–45%; DON, TTX, and T-2 toxins, 60–90%; and FB1, 80–100%. The concentration of OTA, MPA, and FB2 in the infusion depended on its pH. At pH of about 4, it proved to be about 20%, 40%, and 60%, respectively. The increase in infusion pH led to almost complete transfer of these mycotoxins into the solution. The study of naturally contaminated samples supported the results of the model experiments.

**Keywords:** herbal supplements; infusion; mycotoxins; transfer rate; HPLC-MS/MS

### 7.2. Mussel-Based Food Supplements: Evaluation of Emerging Marine Toxins Is a Necessary Evil

OteroPaz*ValeCarmenBoente-JuncalAndreaCostasCeliaLouzaoM. CarmenBotanaLuis M.Departament de Farmacología, Facultad de Veterinaria, Universidad de Santiago de Compostela, 27002 Lugo, Spain*Correspondence: paz.otero@usc.es

Food supplements containing mussel extracts are becoming popular in human diet, providing high levels of proteins, omega-3 polyunsaturated fatty acids (PUFAs), iodine, and carbohydrates. In addition to the beneficial effects and bioactives that mussel may yield, it is vital to consider the potential harmful phycotoxins that can be present in mussel extracts and marine dietary supplements. Recently, we detected for the first time marine toxin 13-desmethyl spirolide C in food supplements containing green lipped mussels *Perna canaliculus* at levels up to 98 µg/kg. In this work, we provided new data of the presence of pinnatoxin-G (trace amounts) in dietary supplements intended for human consumption after the analysis of green lipped mussel powder by ultra-high-performance liquid chromatography coupled with tandem mass spectrometry (UPLC-MS/MS). The status of microalgae phycotoxin contaminants was assessed in these products and in animal dietary supplements that contained 13-desmethyl spirolide C at levels up to 39 µg/kg. The mechanism of action of spirolides and pinnatoxins was associated with the blockage of muscarinic and nicotinic receptors (mAChR and nAChR) in the nervous system. Despite the fact that human intoxications have not been reported, it is important to identify the impact of such toxins on public health, since dietary products constitute an important part of the global market.

**Keywords:** lipophilic toxins; 13-desmethyl spirolide C; pinnatoxin-G; dietary supplements; *Perna canaliculus*; UPLC-MS/MS

### 7.3. Contamination Status of Lipophilic Marine Toxins in Commercial Shellfish from Spain, Chile, and Southeast Pacific

OteroPaz*ValeCarmenBoente-JucalAndreaRaposo-GarcíaSandraCostasCeliaLouzaoM. CarmenBotanaLuis M.Departamento de Farmacología, Facultad de Veterinaria, Universidad de Santiago de Compostela, 27002 Lugo, Spain*Correspondence: paz.otero@usc.es

Lipophilic marine toxins in mollusc constitute an important threat to human health, and a high number of intoxications occur every year. These toxins restrict the progress of aquaculture, which is one of the fastest-growing food sectors in the world. The regions of Galicia (Spain), Chile, and Southeast Pacific are commercially important producers of edible bivalve mollusc; however, they have been subjected to recurring cases of shellfish farm closures in the past decade. This work aimed to study the lipophilic toxic profile of commercial shellfish (including emerging toxins) from these locations in order to establish a potential risk when shellfish are ingested. For this, a total of 41 samples of Galician mussels (*Mytilus galloprovincialis*), 6 samples of mussels from Chile (*Mytilus chilensis*), and 6 samples from Southeast Pacific (*Tawerea gayi* and *Meretrix lyrata*) were purchased from local markets and analyzed by ultra-high-performance liquid chromatography coupled with tandem mass spectrometry (UPLC-MS/MS). Chromatograms from *Mytilus galloprovincialis* showed the presence of okadaic acid (OA), dinophysistoxin-2 (DTX-2), pectenotoxin-2 (PTX-2), azaspiracid-2 (AZA-2), and the emerging toxins 13-desmethyl spirolide C (SPX-13) and pinnatoxin-G (PnTX-G). Data showed that OA group toxins are the main risk in Galician molluscs, which were detected in 38 samples (93%) at levels close to the legislated limit, followed by SPX-13, which was detected in 19 samples (46%) in quantities up to 28.9 μg/kg. Analysis of PTX-2, AZA-2, and PnTX-G showed smaller amounts, all below 3 μg/kg. Results also showed the presence of the emerging PnTX-G in *Mytilus chilensis* at levels up to 5.2 μg/kg and AZA-2 and PTX-2 in the clams *Tawera gayi* up to 4.33 and 10.88 μg/kg, respectively. Although no potential risk through mussel ingestion was found for the emerging toxins (SPX-13 and PnTX-G), there is a need for robust methodologies that can detect a wide range of known or emerging toxins in different matrices due to the geographical expansion of marine toxins.

**Keywords:** lipophilic marine toxins; emerging toxins; *Mytilus galloprovincialis*; *Mytilus chilensis*; *Tawerea gayi*; *Meretrix lyrate*; UPLC-MS/MS

### 7.4. Activation of Anion Channels in Human Cells after Long-Term Exposure to the Marine Toxin Azaspiracid

Boente-JuncalAndreaRaposo-GarcíaSandraCostasCeliaLouzaoM CarmenOteroPazValeCarmen*BotanaLuisDepartamento de Farmacología, Farmacia y Tecnología Farmacéutica, Facultad de Veterinaria, Universidad de Santiago de Compostela, Lugo, Spain.*Correspondence: mdelcarmen.vale@usc.es

Azaspiracids (AZAs) consitute a group of marine toxins first documented in the Netherlands after ingestion of contaminated mussels harvested in Ireland coasts, by the end of the last century [1–3]. Azaspiracids are known to be produced by dinoflagellates belonging to the genera *Azadinium* and *Amphidoma* [4]. In recent years, part of the research on marine toxins’ effects on human health has focused on their chronic effects. The presence of azaspiracid in fishery products has been regulated in Europe, establishing a limit of 160 μg kg^−1^. AZA equivalents [5]. Since then, several acute in vitro studies have been undertaken to elucidate their mechanism of action, but the results obtained show great controversy regarding the possible cellular targets of AZAs that could contribute to the symptomatology elicited in humans after ingestion of contaminated fishery products. Our group recently described that these toxins partially block sodium entry into the cells and cause cytoskeletal alterations [6]; however, the effect of these toxins on ion channels remains almost completely unexplored. Therefore, the main aim of our study was to gain more insight into the effects of azaspiracids on ionic homeostasis and cell volume regulation [7]. Thus, electrophysiological effects of nanomolar concentrations of azaspiracids (50 nM) after a 15–20 h exposure of human embryonic kidney cells (HEK293), which express the human Nav1.7 alpha subunit of the sodium channel, were determined. Here, using electrophysiological techniques combined with several pharmacological approaches, we demonstrated that AZA-1 elicits a significant increase in anion efflux, which could account for the pathophysiology observed in human intoxications.

**Keywords:** azaspiracid; European molluscs; anion channels; voltage-gated chloride channels

**References**

1. McMahon, T. Winter toxicity of unknown aetiology in mussels. *Harmful Algae News* **1996**, *14*, 2.
2. Ofuji, K.; Satake, M.; McMahon, T.; Silke, J.; James, K.J.; Naoki, H.; Oshima, Y.; Yasumoto, T. Two analogs of azaspiracid isolated from mussels, Mytilus edulis, involved in human intoxication in Ireland. *Nat. Toxins* **1999**, *7*, 99–102.
3. Satake, M.; Ofuji, K.; Naoki, H.; James, K.J.; Furey, A.; McMahon, T.; Silke, J.; Yasumoto, T. Azaspiracid, a New Marine Toxin Having Unique Spiro Ring Assemblies, Isolated from Irish Mussels, Mytilus edulis. *J. Am. Chem. Soc.* **1998**, *120*, 9967–9968.
4. Wietkamp, S.; Krock, B.; Clarke, D.; Voß, D.; Salas, R.; Kilcoyne, J.; Tillmann, U. Distribution and abundance of azaspiracid-producing dinophyte species and their toxins in North Atlantic and North Sea waters in summer 2018. *PLoS ONE* **2020**, *15*, e0235015.
5. European Commission. Regulation of the European Parliament and of the Council of 29 April 2004 laying down specific rules for the organisation of official controls on products of animal origin intended for human consumption, 854/2004/EC. *Off. J.* **2004**, *50*.
6. Boente-Juncal, A.; Raposo-García, S.; Costas, C.; Louzao, M.C.; Vale, C.; Botana, L.M. Partial Blockade of Human Voltage-Dependent Sodium Channels by the Marine Toxins Azaspiracids. *Chem. Res. Toxicol.* **2020**, doi:10.1021/acs.chemrestox.0c00216.
7. Vale, C.; Nicolaou, K.C.; Frederick, M.O.; Vieytes, M.R.; Botana, L.M. Cell volume decrease as a link between azaspiracid-induced cytotoxicity and c-Jun-N-terminal kinase activation in cultured neurons. *Toxicol. Sci.* **2010**, *113*, 158–168.

### 7.5. Neuropeptide Y’s Protective Role in Okadaic-Acid-Induced Diarrhea

CostasCelia^1^LouzaoM. Carmen^1^*AbalPaula^1^OteroPaz^1^Boente-JuncalAndrea^1^ValeCarmen^1^VilariñoNatalia^1^VieytesMercedes R.^2^BotanaLuis M.^1^1Departamento de Farmacología, Facultad de Veterinaria, Universidad de Santiago de Compostela, 27002 Lugo, Spain2Departamento de Fisiología, Facultad de Veterinaria, Universidad de Santiago de Compostela, 27002 Lugo, Spain*Correspondence: mcarmen.louzao@usc.es

Marine biotoxins represent a major threat to public health. Microalgae, such as diatoms or dinoflagellates, are major producers of these compounds. Blooms of these species commonly correspond with increased toxin concentrations in filter-feeding organisms, which can lead to poisoning outbreaks due to shellfish consumption. Within toxins of marine origin, we focus our work on the okadaic acid (OA) group, which also includes the analogues dynophysistoxins 1 and 2. Diarrheic shellfish poisoning (DSP) develops after ingestion of contaminated food containing these lipophilic compounds, involving mainly gastrointestinal symptoms like nausea and diarrhea. The OA group of toxins inhibits protein phosphatases (PPs) with ubiquitous distribution, like PP1 or PP2A. Yet, several reports have raised the possibility of the phycotoxin targeting different mechanisms. A substantial variety of pathogenic stimuli trigger diarrhea through the activation of the enteric nervous system (ENS). Neuropeptide Y (NPY) is a 36-amino-acid peptide of neuronal origin known to maintain an antisecretory tone by acting on the receptors Y_1_ and Y_2_, both expressed along the gastrointestinal tract. Previous in vitro studies have exposed that OA downregulates the NPY gene and protein expression. Moreover, the toxin is reported to cause diarrhea within the first 2 h of treatment. Here, we assess how the pro-absorptive NPY could modify OA-induced diarrhea in vivo. Mice were first given NPY intraperitoneally 15 min prior to OA oral gavage administration. Body weight variations, symptoms, and food and water intake were monitored after 2 h of treatment. Afterward, anatomopathological examination took place and intestine samples were collected for transmission electron microscopy evaluation. In the presence of NPY, no delay in diarrhea onset was observed, though ultrastructural mild recovery was detected in the large intestine. Hence, it could be feasible that OA modifies the NPY antisecretory tone, resulting in diarrhea.

**Keywords:** neuropeptide Y; okadaic acid; diarrheic shellfish poisoning (DSP)

### 7.6. Detection of Ciguatoxins in Fish and Algal Samples with an Electrochemical Biosensor

GaianiGreta^1^*LeonardoSandra^1^TsumurayaTakeshi^2^RamblaMaria^1^DiogèneJorge^1^O’SullivanCiara Kathleen^3^,^4^AlcarazCarles^1^CampàsMònica^1^*1IRTA, Ctra Poble Nou km 5.5, 43540 Sant Carles de la Ràpita, Spain2Department of Biological Science, Graduate School of Science, Osaka Prefecture University, 1-2, Gakuen-cho, Naka-ku, Sakai, Osaka 599-8570, Japan3Departament d’Enginyeria Química, URV, Països Catalans 26, 43007 Tarragona, Spain4ICREA, Pg. Lluís Companys 23, 08010 Barcelona, Spain*Correspondence: greta.gaiani@irta.cat (G.G.); monica.campas@irta.cat (M.C.)

Ciguatera fish poisoning (CFP) is one of the most relevant seafood-borne diseases worldwide. It is caused by the ingestion of fish containing ciguatoxins (CTXs), lipophilic marine toxins produced by microalgae of the genera *Gambierdiscus* and *Fukuyoa* that accumulate into fish flesh and through the food webs. CFP is characterized by severe neurological, gastrointestinal, and cardiovascular disorders and affects approximately between 50,000 and 500,000 consumers annually worldwide. The real incidence of CFP is difficult to ascertain due to under-reporting and misdiagnosis. Here, the first electrochemical immunosensor for the detection of CTXs is presented. Three different monoclonal antibodies (mAbs), two capture (3G8, 10C9) and a detector (8H4), were merged in a sandwich configuration for the combined detection of two main groups of CTX congeners (CTX1B and CTX3C). Initially, the applicability of the immunosensor was demonstrated with the analysis of fish samples coming from La Réunion Island, providing results that correlate with the mouse bioassay and cell-based assay. Then, fish coming from Mediterranean waters were analyzed, with promising results. Finally, extracts from *Gambierdiscus * and *Fukuyoa* were screened, allowing the separate detection of the two groups of CTX congeners and providing new information regarding the toxin production of the genera. The developed bioanalytical tool is user friendly and can help to mitigate ciguatera risk, contributing to the protection of consumer health.

**Keywords:** ciguatera; ciguatoxin; electrochemical immunosensor; Gambierdiscus

### 7.7. Paretic Syndrome in Gulls from Southern Portugal: Searching for the Causative Agent

Ben-GigireyBegoña^1^*MenaMaría V.^2^MazuetChristelle^3^RiobóPilar^4^RodríguezFrancisco^5^1Instituto Español de Oceanografía (IEO), Centro Oceanográfico de Vigo, Subida a Radio Faro, 50, 36390 Vigo, Spain2RIAS Wildlife Rehabilitation and Research Centre, Parque Natural da Ria Formosa, 8700-194 Olhão, Portugal3Pasteur Institute, CNR Bactéries anaérobies et Botulisme, 25 rue du Dr Roux 75015 Paris, France4Instituto de Investigaciones Marinas, Consejo Superior de Investigaciones Científicas (IIM-CSIC), 36208, Vigo, Spain5Instituto Español de Oceanografía (IEO), Centro Oceanográfico de Vigo, 36390, Vigo, Spain*Correspondence: begona.ben@ieo.es

Between 2010 and 2019, 2432 gulls (Larus michahellis and Larus fuscus) with paretic syndrome were received at the RIAS Wildlife Rehabilitation and Research Centre. The clinical signs included weakness, anorexia, paralysis, diarrhea (flaccid cloacae), dyspnea, and, in some cases, death. Several biotic contaminants are among the potential causes of this syndrome: marine biotoxins, Clostridium botulinum, cyanotoxins, and virus. This presentation compiles the results of the Clostridium botulinum and marine biotoxin analysis conducted at the French National Reference Centre for anaerobic bacteria and botulism, the Pasteur Institute (Paris), and the Vigo Centre of the Spanish Oceanographic Institute. C. botulinum analyses were conducted in livers and intestines from five gulls with paretic syndrome symptoms admitted at the RIAS Wildlife Rehabilitation and Research Centre. Samples were pooled in two groups according to tissue and analyzed by targeted real-time polymerase chain reaction (PCR) on neurotoxin genes after sample enrichment culture under anaerobic conditions. The presence of botulinum toxin was confirmed by a lethality test on mice (mouse bioassay). Mice were intraperitoneally injected with the filtered supernatant of the culture. Paralytic shellfish toxins (PSTs) were analyzed by liquid chromatography with fluorescence detection and post-column oxidation in samples from 10 gull kidneys and in the cloacae contents from another gull. Domoic acid (DA) analysis was conducted following a procedure that involved methanolic extraction and analysis by liquid chromatography coupled with high-resolution mass spectrometry. DA was analysed in 23 gull samples: 10 livers, 10 intestines, and 3 cloacae. PSTs and DA were not detected in any of the samples tested. Results obtained so far point to C. botulimum type C/D as the causative agent of paretic syndrome in gulls.

**Keywords:** paralytic shellfish toxins; domoic acid; *Clostridium botulinum*; seagulls; paretic syndrome

### 7.8. Recombinase Polymerase Amplification for Gambierdiscus and Fukuyoa Detection: A Step Further in Ciguatera Risk Management

GaianiGreta^1^*ToldràAnna^1^ReyMaria^1^AndreeKarl^1^AlcarazCarles^1^DiogèneJorge^1^O’SullivanCiara Kathleen^2^,^3^CampàsMònica^1^*1IRTA, Ctra Poble Nou km 5.5, 43540 Sant Carles de la Ràpita, Spain2Departament d’Enginyeria Química, URV, Països Catalans 26, 43007 Tarragona, Spain3ICREA, Pg. Lluís Companys 23, 08010 Barcelona, Spain*Correspondence: greta.gaiani@irta.cat (G.G.); monica.campas@irta.cat (M.C.)

Ciguatera fish poisoning is one of the most relevant seafood-borne illnesses worldwide. It is caused by the ingestion of fish contaminated by ciguatoxins (CTXs). Primary producers of CTXs are dinoflagellates of the genera Gambierdiscus and Fukuyoa. This study focuses on the development of bioanalytical tools for the detection of Gambierdiscus and Fukuyoa. To achieve this objective, recombinase polymerase amplification (RPA), which consists of isothermal DNA amplification during a short period (30 min), was combined with enzyme-linked oligonucleotide assay (ELONA). To evaluate the specificity of RPA-ELONA, first primers for the genera Gambierdiscus/Fukuyoa were exposed to genomic DNA of different species (G. australes, G. excentricus, G. belizeanus, G. balechi, and F. paulensis) and other microalgae used as controls (O. cf. ovata, P. lima, and C. monotis). The same genomic DNA pools were also tested with species-specific primers for Gambierdiscus australes and Gambierdiscus excentricus. Finally, DNA was extracted from single cells of the previously mentioned genera and species and tested with all the primer sets. For both experiments, detection was achieved only when combining capture probes with their target RPA product, and no significant responses were observed in the presence of non-target DNA. Obtained results demonstrate the ability of the system to discriminate not only the genus Gambierdiscus/Fukuyoa from other microalgae but also G. australes and G. excentricus species from their congeners. Furthermore, the limit of detection is as low as a single cell.

**Keywords:** ciguatera; *Gambierdiscus*; *Fukuyoa*; recombinase polymerase amplification

**Acknowledgments:** The research received funding from the Ministerio de Ciencia, Innovación y Universidades, through the CIGUASENSING project (BIO2017-87946-C2-2-R). The authors acknowledge support from the CERCA Programme/Generalitat de Catalunya. G. Gaiani acknowledges the IRTA-Universitat Rovira i Virgili-Banco Santander for her PhD grant (2018PMF-PIPF-19).

### 7.9. Cyclodextrins as Capture Agents of Lipophilic Marine Toxins

WirénCharlotta^1^,^2^*Rambla-AlegreMaria^1^SafontAnna^1^AlcarazCarles^1^DiogèneJorge^1^TorrénsMabel^3^FragosoAlex^3^CampàsMònica^1^*1IRTA, Ctra Poble Nou km 5.5, 43540 Sant Carles de la Ràpita, Spain2UAB, Bellaterra, 08193 Barcelona, Spain3Departament d’Enginyeria Química, URV, Països Catalans 26, 43007 Tarragona, Spain*Correspondence: wiren.charlotta@gmail.com (C.W.); monica.campas@irta.cat (M.C.)

Seafood contamination by marine toxins from harmful algal blooms (HABs) is a global public health issue on the rise. Most countries have monitoring programs in place for the detection of toxins in shellfish of toxic phytoplankton in seawater to prevent consumer intoxications. The use of the solid-phase adsorbent- and toxin-tracking (SPATT) technology for toxin detection straight from the aquatic environment could complement the labor-intensive traditional monitoring methods. In this work, several types of cyclodextrins (cyclic oligomers with a conical structure and an internal cavity) were evaluated as novel materials for SPATT. Cyclodextrins were tested at the Masnou harbor (Catalonia, northwest Mediterranean) during a *Dinophysis* sp. bloom. The cyclodextrins and the commercial Diaion (HP-20) were deployed twice for a 1-week period at five different locations of the Masnou harbor. At the time of the experiment, *Dinophysis* sp. reached abundances as high as 91,341 cells/L. Successful accumulation of lipophilic marine toxins (okadaic acid (OA) and pectenotoxin-2 (PTX2)) was quantified by liquid chromatography–tandem mass spectrometry (LC-MS/MS). Higher levels of PTX2 were found in all cyclodextrins, whereas OA and PTX2 contents were similar in the commercial resin. Accumulation of OA was higher in the commercial resin than in cyclodextrins, but these proved best for PTX2 adsorption. A clear correlation between cell abundance and toxin accumulation was observed.

**Keywords:** Cyclodextrins; Lipophilic toxins; Harmful algal bloom (HAB); Solid-phase adsorption toxin tracking (SPATT); Liquid chromatography-tandem mass spectrometry (LC-MS/MS)

**Acknowledgments:** The research received funding from the Ministerio de Ciencia, Innovación y Universidades (MICINN), the Agencia Estatal de Investigación (AEI), and the Fondo Europeo de Desarrollo Regional (FEDER) through the CIGUASENSING project (BIO2017-87946-C2-1-R and BIO2017-87946-C2-2-R). The authors acknowledge support from the CERCA Programme/Generalitat de Catalunya.

### 7.10. Smartphone-Based Electrochemical Immunosensor for Ciguatoxin Detection

LeonardoSandra^1^*TsumurayaTakeshi^2^OshiroNaomasa^3^HiramaMasahiro^2^DiogèneJorge^1^CampàsMònica^1^1IRTA, Ctra Poble Nou km 5.5, 43540 Sant Carles de la Ràpita, Spain2Department of Biological Sciences, Graduate School of Science, Osaka Prefecture University, Osaka 599-8570, Japan3National Institute of Health Sciences, 3-25-26 Tonomachi, Kawasaki, Kanagawa 210-9501, Japan*Correspondence: sandra.leonardo@irta.cat

Ciguatera fish poisoning (CFP) is the most common and one of the most relevant seafood-borne diseases worldwide. CFP is caused by the ingestion of fish contaminated by ciguatoxins (CTXs), potent lipophilic marine toxins with complex chemical structures produced by microalgae of the genera *Gambierdiscus* and *Fukuyoa*, which are transferred and metabolized through the food webs. The importance of CTXs in seafood safety and their emerging occurrence in locations far away from the tropical areas where they have been historically found highlight the need for alternative analytical methods for their rapid, simple, and cost-effective detection. In this sense, a portable electrochemical biosensor for the detection of CTXs is presented. Two different capture antibodies able to recognize the left wing of CTX1B and 54-deoxyCTX1B and the left wing of CTX3C and 51-hydroxyCTX3C were immobilized on multi-walled carbon-nanotube-modified electrodes. A sandwich configuration was adopted by the use of a biotinylated antibody that binds to the right wing of these four CTX congeners. PolyHRP-streptavidin was used as an enzymatic label for signal amplification and detection of the biotinylated antibody. Amperometric currents werre recorded with a small and ready-to-go potentiostat inserted in a smartphone, providing in situ measurements. A CTX1B calibration curve was obtained, achieving a limit of detection at the pg/mL level. After the evaluation of matrix effects, the ability of the immunosensor to detect CTX1B contents at the 0.01 µg/kg guidance level proposed by the United States Food and Drug Administration (US FDA) was demonstrated. The biosensor is being applied to the analysis of naturally contaminated fish samples, and results will be compared with those obtained by cell-based assay (CBA) and liquid chromatography coupled with mass spectrometry (LC/MS). This portable, easy-to-handle, rapid, and low-cost analytical tool will facilitate the monitoring of CTX contents to guarantee seafood safety.

**Keywords:** ciguatoxins; electrochemical immunosensor; smartphone; portable; fish

### 7.11. Scorpionic Serotherapy in Pregnancy and Its Effects on the Offspring

PazGuilherme Gonelli^1^,^2^*NencioniAna Leonor Abrahão^1^1Laboratory of Pharmacology, Butantan Institute, São Paulo, Brazil2Pos Graduation Program on Toxinology, Butantan Institute, São Paulo, Brazil*Correspondence: guilherme.paz@butantan.gov.br

Scorpionic poisoning is a public health problem due to the high number of cases registered not only in Brazil but also in the world, mainly in tropical and subtropical areas. It is known that scorpion poisoning can cause problems ranging from simple local manifestations, such as small edemas, to serious problems, such as cardiocirculatory complications, which can lead to death. In the case of poisoning of women during pregnancy, there are risks for both the mother and the fetus, causing the death of both in extreme cases. In previous studies, we observed that when the venom of the scorpion Tityus bahiensis is administered to rats during pregnancy or lactation, changes in the physical, reflexological, and behavioral development of the offspring occur, both in the perinatal phase and during adulthood, as well as changes in the levels of some cytokines and neurotransmitters. Serotherapy is the most suitable method for treating scorpion poisoning. However, there are few studies regarding the effects that antivenom can have on the fetus, whether beneficial or otherwise. Therefore, this project aims to study and elucidate the effects of perinatal scorpion serotherapy, checking whether there is any physiological change in the fetus, as well as whether there is a reversal of the changes caused by the poisoning of the mother.

**Keywords:** poisoning; pregnancy; offspring; scorpion; neurotoxin

## 8. Impact of Toxins on Agriculture

### 8.1. Study of the Influence of Picking Frequency and Drying Materials on Mold and Aflatoxin Occurrence in Cashew Nuts from Côte d’Ivoire

StephaneKoffi Yao^1^HalbinKouadio James^1^,^2^*1Research Group of Crop Production Quality Management, Laboratory of Agrovalorisation, UFR Agroforesterie, Jean Lorougnon Guédé University, BP 150 Daloa, Côte d’Ivoire2Laboratory of (Bio) Toxicology and Industrial Hygiene, DPPSST, CNPS 01 B.P. 317 Abidjan, Côte d’Ivoire*Correspondence: jameshalbink@yahoo.fr

The aim of the present study was to evaluate the impact of picking frequency and drying materials on fungal infection and the aflatoxin content in cashew nuts produced in Côte d’Ivoire. Some cashew nuts collected after a delay of 2, 3, and 7 days on the ground were sun-dried on a rack-tables, tarpaulins, and cemented areas until the moisture content reached 8%. After 6 months of storage, 18 samples (3 kg of each) of cashew nuts were collected and fungal infection and the aflatoxin content were evaluated using standard methods. Our results revealed that the rate of fungal infection evolved in keeping with the frequency of nut picking. Thus, the rate of fungal infection was 5.7% at 2 days of frequency of nut picking, 22.7% at 3 days, and 54.6% at 7 days. Proportionally, the loss rates were 1.85%, 4.73%, and 11.03%, respectively. The dryers had no significant effect on the infection and loss rates, with corresponding values ranging from 24.49% to 31.45% and 5.41% to 10.32%, respectively. A total of 12 genera and 148 fungal species were isolated and identified. The genus Aspergillus represented by Aspergillus niger (71.78%), *Aspergillus flavus* (4.29%), Aspergillus fumigatus (2.45%), and Aspergillus sp (1.84%) was the most preponderant. Although aflatoxin levels were marginal, they reached 0.34 µg/kg with the 7-day pickup time. This aflatoxin level is related to the presence of aflatoxin B1 (0.29 µg/kg). The drying supports, namely tarpaulins or cemented areas, seemed to influence the level of aflatoxin secretion. Taken together, our results suggest that the frequency of cashew nut picking is a critical control point in the value chain for both fungal infection and aflatoxin contamination. In a certain way, the rack-table seems a suitable drying support to avoid aflatoxin contamination.

**Keywords:** cashew nuts; picking frequency; dryers; *Aspergillus* sp.; aflatoxins

### 8.2. Quantitative Determination of Aflatoxin B1 Levels in Rice Grans Using an Enzyme-Linked Immunosorbent Assay-Validated Method in Kenya

DouksounaYoumma^1^*TonuiRonald^2^NyerereAndrew^1^,^3^RunoSteven^1^,^4^AmbangZachée^5^1Department of Molecular Biology and Biotechnoly, Pan African University Institute for Basic Sciences Technology and Innovation, P.O. Box 62000-00200 Nairobi, Kenya2Department of Biochemistry and Microbiology, Rhodes University-South Africa3Department of Medical Microbiology, Jomo Kenyatta University of Agriculture and Technology, P.O. Box 62000-00200 Nairobi, Kenya4Department of Biochemistry, Microbiology and Biotechnology, Kenyatta University, P.O. Box 43844-00100 Nairobi, Kenya5Department of Plant Biology and Biotechnology, University of Yaounde1, P.O. Box 812 Yaoundé, Cameroon*Correspondence: ydouksouna@gmail.com

Aflatoxins are secondary metabolites produced by *Aspergillus* species distributed on three main sections of the genus, namely section *A. Flavi*, section *A. Ochraceorosei*, and section *A. Nidulantes*. They are common contaminates of dietary staples worldwide, including cereals, oil seeds, nuts, spices, meats, dairy products, fruit juices, dried fruits, eggs, and feeds and foods derived from these products. Aflatoxins are unavoidable widespread natural contaminants of foodstuffs, with serious impacts on food safety, health, and agricultural and livestock productivity. Aflatoxin B_1_ is the analyte with the highest toxic significance and the most potent hepatocarcinogenic among other aflatoxins, and humans may get exposed to it at any stage of life. Dietary exposure to aflatoxins is a public health concern due to their carcinogenic, acute aflatoxicosis, and chronic effects and immunosuppression properties, among others. This study focused on aflatoxin B_1_ in rice grains. Rice is an important staple food consumed widely and comprises a major part of the diet for half of the world population. In general, there have been few reports on the occurrence of aflatoxin B_1_ in rice grains compared to other cereals in Africa. However, aflatoxin B_1_ levels in rice grains compared to other crops are of concern because of the high consumption of rice in several countries in Africa. This study assessed aflatoxin B_1_ in rice grains: its occurrence, control, and socioeconomic and health implications. We quantitatively determined the levels of aflatoxin B_1_ using enzyme-linked immunosorbent assay. Of all examined samples, 43.1% were positive (15.9% local rice and 27.2% imported rice) and 11.3% were above the maximum limit of aflatoxin B_1_ in rice established by the European Union. According to the manufacturer’s instructions, the limit of detection is 1 μg/kg (ppb) in cereals. The concentration of aflatoxin B_1_ in examined samples ranged from 0 to 3.2 μg/kg. These results are indicative of exposure of the population to aflatoxin and the possible health hazard. The procedure used in this study is suitable for detection of mycotoxins at a low concentrations.

**Keywords:** rice; contamination; aflatoxins; occurrence; incidence; exposure; food; safety

**Key Contribution:** This article provides insight into the contamination of rice grains by aflatoxin B1, as rice is a staple food in Africa. It assesses aflatoxin B1 in rice grains and its occurrence, control, and socioeconomic and health implications in order to ensure food safety.

### 8.3. Determination and Occurrence of Ergot Alkaloids in Cereal Samples from Algeria

Carbonell-RozasLaura^1^*MahdjoubiChoukri Khelifa^2^Arroyo-ManzanaresNatalia^3^Gámiz-GraciaLaura^1^García-CampañaAna M.^1^1Department of Analytical Chemistry, Faculty of Sciences, University of Granada, Avda. Fuente Nueva s/n, 18071, Granada, Spain2Department Biology, Faculty of Natural and Life Science, University of Oran 1, Algeria3Department Analytical Chemistry, Faculty of Chemistry, University of Murcia, 30100 Murcia, Spain*Correspondence: rozas@ugr.es

Mycotoxins are fungal secondary metabolites naturally present in different food and feed, with toxic effects to humans and animals that consume these contaminated products. Among them, ergot alkaloids (EAs), produced mainly by fungi of the *Claviceps* genus, such as *Claviceps purpurea*, are present in cereals such as rye, triticale, wheat, and barley. Improvements in agricultural practices have significantly reduced the risk of severe epidemic outbreaks of ergotism; however, EAs can be found in cereal-based food and feed, partially due to new cereal hybrids susceptible to *C. purpurea* and climate change. The European Commission has established a maximum content of 0.5 g/kg of ergot sclerotia in unprocessed cereals (with the exception of corn and rice), but the maximum content for EAs is still under study. Moreover, other countries (such as Algeria) have no legislation regarding mycotoxin contamination. In this study, the major EAs (ergometrine (Em), ergosine (Es), ergotamine (Et), ergocornine (Eco), ergokryptine (Ekr), and ergocristine (Ecr)) and their corresponding epimers (ergometrinine (Emn), ergosinine (Esn), ergotaminine (Etn), ergocorninine (Econ), ergokryptinine (Ekrn), and ergocristinine (Ecrn)) were determined by the QuEChERS–ultra-high-performance liquid chromatography coupled with tandem mass spectrometry (UHPLC-MS/MS) method in cereal samples from Algeria (30 samples of wheat and 30 samples of barley). Procedural calibration curves were established for both matrices, and limits of quantification were below 3.3 μg/kg for wheat and 3.9 μg/kg for barley. The recoveries ranged between 85% and 109%, with a matrix effect lower than 20% in most cases and precision (relative standard deviation (RSD)) lower than 11%. Four barley samples were contaminated by Em and Emn, and 3 of them also showed contamination by Et, with the total EA content ranging from 18.0 to 54.0 μg/kg. Wheat samples showed higher contamination, with eight positive samples: one sample was only contaminated by Em, while the rest were contaminated by 5 to 11 EAs, with the total EA content ranging from 6.5 to 77.4 μg/kg.

**Keywords:** ergot alkaloids; liquid chromatography; cereal samples

### 8.4. Validation of a Method for the Control of Ergot Alkaloids in Oat-Based Functional Foods

Carbonell-RozasLaura*LaraFrancisco J.Gámiz-GraciaLauraGarcía-CampañaAna M.Department of Analytical Chemistry, Faculty of Sciences, University of Granada, Avda. Fuente Nueva s/n, 18071 Granada, Spain*Correspondence: rozas@ugr.es

Ergot alkaloids (EAs) are mycotoxins produced mainly by fungi of the Claviceps genus, such as *Claviceps purpurea*. The fungus infects the seed heads of living plants, especially cereals, at the time of flowering, replacing the developing grain or seed with specialized fungal structures known as the sclerotium (or ergot body), which contains alkaloid substances. More than 50 different EAs have been identified, the major compounds being ergometrine (Em), ergosine (Es), ergotamine (Et), ergocornine (Eco), ergokryptine (Ekr), ergocristine (Ecr), and their corresponding epimers, ergometrinine (Emn), ergosinine (Esn), ergotaminine (Etn), ergocorninine (Econ), ergokryptinine (Ekrn), and ergocristinine (Ecrn). Although sclerotia can be mechanically removed during the harvesting process, EAs can be found in cereal-based food and feed, and their ingestion might cause adverse health effects in humans and animals. The European Commission has established a maximum content of 0.5 g/kg of ergot sclerotia in most unprocessed cereals; however, the maximum content of EAs allowed in food is still under study. In this work, we propose the extraction and quantification of the main EAs and their epimers in different oat-based products by QuEChERS extraction followed by ultra-high-performance liquid chromatography coupled with tandem mass spectrometry (UHPLC-MS/MS). The recoveries ranged between 89% and 106%, with a matrix effect lower than 20% in most cases and precision (intra- and interday), expressed as the relative standard deviation (RSD), lower than 15%. Procedural calibration curves were established, and limits of detection and quantification were below 1.0 and 3.2 μg/kg, respectively. Finally, 25 oat-based samples (including bran, flakes, juices, hydroalcoholic extracts, flours, tablets, and grass) were analyzed. One of the samples of oat bran was contaminated by Em, Emn, Es, and Esn in the range of 1.1–7.2 μg/kg, with a total EA content of 10.7 μg/kg.

**Keywords:** ergot alkaloids; functional foods; liquid chromatography; tandem mass spectrometry

**Acknowledgments:** Financial support of the Spanish Ministry of Science, Innovation and Universities (Project Ref.: RTI2018-097043-B-I00) is acknowledged.

### 8.5. Occurrence of Principal Ergot Alkaloids in Swine Feeding

Gámiz-GraciaLaura^1^*Arroyo-ManzanaresNatalia^2^Rodríguez-EstévezVicente^3^García-CampañaAna M.^1^1Department Analytical Chemistry, Faculty of Sciences, University of Granada, Spain2Department Analytical Chemistry, Faculty of Chemistry, University of Murcia, Spain3Department Animal Production, Faculty of Veterinary, University of Córdoba, Spain*Correspondence: lgamiz@ugr.es

Ergot alkaloids (EAs) are secondary metabolites produced by fungi of the genus Claviceps that contaminate a large variety of cereals. More than 50 different EAs have been identified, the major compounds being ergometrine (Em), ergosine (Es), ergotamine (Et), ergocornine (Eco), ergokryptine (Ekr), ergocristine (Ecr), and their corresponding epimers, ergometrinine (Emn), ergosinine (Esn), ergotaminine (Etn), ergocorninine (Econ), ergokryptinine (Ekrn), and ergocristinine (Ecrn). The ingestion of contaminated cereals might cause adverse health effects in humans and animals, such as the well-known ergotism. In fact, pigs, cattle, sheep, and poultry are involved in sporadic outbreaks, although most other species are also susceptible. EAs’ toxicity is linked to their structural similarity with dopamine, noradrenaline, adrenaline, and serotonin, enabling binding to the biogenic amine receptor and the interruption of neurotransmission. The European Union (EU) has established a maximum content of 1000 mg/kg of rye ergot sclerotia (*Claviceps purpurea*) in feed materials and compound feed containing unground cereals. Although EAs as such are still not regulated, the feed industry recommends practical limits for EAs in pig feed to reduce negative effects on health and performance. However, the absence of sclerotia does not exclude the presence of EAs. In this work, 12 EAs were quantified in 228 feed samples intended for swine using QuEChERS as sample treatment and ultra-high-performance liquid chromatography coupled with tandem mass spectrometry (UHPLC-MS/MS) for determination. Of the samples, 12.7% (29 samples) revealed contamination by at least one EA, and among the contaminated samples, 65% were contaminated by more than one EA. Only 6 of 12 target EAs showed concentrations above the limit of quantification. The highest concentration was detected for Emn (with concentrations up to 145 μg/kg), while the total EA content ranged from 5.9 to 158.7 μg/kg. This study revealed scarce contamination of Spanish feed samples by EAs.

**Keywords:** ergot alkaloids; feed; pig; LC-MS/MS

### 8.6. Impaired Broiler Performance and Intestinal Integrity as a Carry-Over Effect in Broilers Fed Diets Naturally Contaminated by Moderate Levels of Deoxynivalenol

SantosRegiane*EerdenEllen vanDepartment of Research and Development, Schothorst Feed Research, Meerkoetenweg 26, 8218 NA Lelystad, The Netherlands*Correspondence: rsantos@schothorst.nl

Natural exposure to mycotoxins is a common event in the poultry industry. Deoxynivalenol (DON) is usually detected at levels lower than the maximum recommended ones (5000 ppb). However, depending on the diet and bird age, such low levels might be sufficient to induce intestinal damage and impair broiler performance. We evaluated the effect of 900 and 2300 ppb DON with or without activated charcoal as a binding agent on performance and intestinal morphometry and lesions in broilers. We divided 736-day-old male Ross broilers (*n* = 308) into four treatments with eight replicates. The broilers were fed diets naturally contaminated by low DON (LD; 900 ppb) or moderate DON (MD; 2300 ppb) with or without activated charcoal for 28 days. Afterward, all birds were fed a diet without DON or activated charcoal for 7 days. During the first 28 days of the trial, MD diet without activated charcoal significantly reduced body weight gain and the FCR. Even after the 7-day wash-out period, MD diet resulted in an overall significantly reduced body weight gain and FCR regardless of the presence of activated charcoal. At 28 days, MD diet without activated charcoal caused a decrease in the jejunum villus height and an increase in the ileum crypt depth, thereby reducing the villus:crypt ratio in both intestinal segments. Based on these results, it can be concluded that broiler production and intestinal morphology are negatively affected when feed is contaminated by DON, even at moderate levels (2300 ppb), and performance losses are not recovered even if the broilers are fed a non-contaminated diet afterward.

**Keywords:** broiler; deoxynivalenol; activated charcoal; performance; intestinal morphology

### 8.7. Flavonoids Play a Key Role in Resistance to Accumulation of Aflatoxin in Corn

Castano-DuqueLina*MackBrian MGilbertMatthew KSicklerChristine MCaryJeffrey WRajasekaranKanniahUSDA-ARS, USA*Correspondence: Lina.Castano.Duque@usda.gov

*Aspergillus flavus* is a facultative pathogen capable of producing aflatoxins (AF), potent carcinogens that accumulate in corn kernels, peanuts, cottonseed, and tree nuts. To understand resistance mechanisms to AF accumulation in corn, we performed a high-throughput genomics study using an in vitro kernel screening assay with *A. flavus* 3357, resistant corn hybrid TZAR102 and, susceptible corn hybrid Va35. Furthermore, we incorporated gene expression data with genomic data to perform redundancy analysis (RDA). We determined that the corn genotype, fungal treatment, and duration of infection significantly co-vary to influence overall gene expression patterns. We performed gene ontology enrichment analysis on highly significant genes and found the enrichment of pathways linked to fungal and microbial responses, such as pathogenesis-related (PR) proteins. To determine additional genes of interest using field and gene expression data, we linked genome-wide association analysis results with gene expression data, allowing us to detect significant expression quantitative trait loci (eQTL). Our results showed that resistance to aflatoxin contamination is a polygenic trait, and we found significant association between specific flavonoid biosynthetic pathway genes and infection by *A. flavus*. Additional experiments including functional genomics analyses and fungal bioassays to identify the role of flavonoids and their contribution to corn resistance to *A. flavus* growth and AF production was also presented.

**Keywords:** aflatoxin; corn; *Aspergillus*; GWAS; flavonoids

### 8.8. Genetic Responses and Aflatoxin Inhibition during Interaction between Aflatoxigenic and Non-Aflatoxigenic Aspergillus flavus

SweanyRebecca Ruth^1^,^2^*MackBrian M^2^MooreGeromy G^2^GilbertMatthew K^2^CaryJeffrey W^2^RajasekaranKanniah^2^DamannKenneth EJr.1Louisiana State University, Baton Rouge, LA, USA2U.S. Department of Agriculture-Southern Regional Research Center, New Orleans, LA, USA*Correspondence: rebecca.sweany@usda.gov

Aflatoxin is a carcinogenic mycotoxin produced by *Aspergillus flavus* in corn. Non-aflatoxigenic *A. flavus* isolates are applied to corn fields as a biocontrol to reduce aflatoxin contamination. Direct contact or touch between aflatoxigenic and non-aflatoxigenic isolates dramatically reduces aflatoxin production. To understand the mechanism of touch inhibition, a high-throughput RNA-seq study was conducted to examine gene expression during their interaction. The non-aflatoxigenic strain KD17 and the aflatoxigenic strain KD53 were grown separately and in co-culture for 30 and 72 h. Toxin production was high in the aflatoxigenic monoculture and negligible in co-cultures. When grown separately, the toxigenic strain represented 7% and 33% of the combined biomass at 30 and 72 h, respectively. However, only 3% of the sequence reads uniquely aligned to the aflatoxigenic strain during co-culture, indicating growth and/or gene expression of the aflatoxigenic strain was inhibited in response to the non-aflatoxigenic strain. Few reads aligned to the aflatoxin gene cluster during co-culture. Eighteen genes expressed during monoculture of the non-aflatoxigenic strain were further upregulated during co-culture, indicating a response to contact. Of those genes, seven belong to a putative secondary metabolite cluster, suggesting a potentially inhibitory compound is produced. Taken together, these results suggest that non-aflatoxigenic strains inhibit growth and aflatoxin biosynthetic gene cluster expression in aflatoxin-producing strains. In addition, other secondary metabolite genes are upregulated during biocontrol interaction. This study demonstrates the potential role of inhibitory secondary metabolites in the biocontrol mechanism and deserves further exploration to improve biocontrol formulations.

**Keywords:** biocontrol; aflatoxin; toxin inhibition; RNA-seq; corn

## 9. Evolution of Toxins

### 9.1. Functional Role of Individual Parts of Bacillus cereus Hemolysin II

SiunovAlexander V^1^NagelAlexey S^1^Andreeva-KovalevskayaZhanna I^1^ZamyatinaAnna V^2^RudenkoNatalia V^2^,^3^KaratovskayaAnna P^2^BorisovMarina P^4^SalyamovVadim I^1^KolesnikovAlexander S^1^,^3^MelnikBogdan S^5^BrovkoFedor A^2^,^3^SoloninAlexander S^1^*1FSBIS FRC Pushchino Scientific Centre of Biological Research, G.K. Skryabin Institute of Biochemistry and Physiology of Microorganisms, Russian Academy of Sciences, 5 Prospekt Nauki, 142290 Pushchino, Moscow Region, Russia2Pushchino Branch, Shemyakin– Ovchinnikov Institute of Bioorganic Chemistry, 6 Prospekt Nauki, 142290 Pushchino, Moscow Region, Russia3Pushchino State Institute of Natural Sciences, 3 Prospekt Nauki, 142290 Pushchino, Moscow Region, Russia4Institute of Theoretical and Experimental Biophysics RAS, 2 Prospekt Nauki, 142290 Pushchino, Moscow region, Russia;5Protein Institute of the Russian Academy of Sciences, 4 Prospekt Nauki, 142290 Pushchino, Moscow Region, Russia*Correspondence: solonin.a.s@yandex.ru

Hemolysin II of *Bacillus cereus* sensu lato is synthesized in a bacterial cell in the form of a water-soluble secreted monomer and penetrates into eukaryotic membranes. The HlyII protein has a C-terminal extension (HlyIICTD) and includes 94 amino acid residues [1]. Removal of HlyIICTD from HlyII significantly complicates the transfer of the deletion variant HlyIIDCTD to E. coli cells, possibly due to the attack of the bacterial membrane. Additional deletion of the signal peptide, which excludes the penetration of the protein into the periplasm, provides E. coli cells with survival when carrying this gene with two deletions. Using monoclonal antibodies against recombinant HlyIICTD, Rudenko et al. [2] showed a similar effective binding to red blood cells of various origins and noticeably different for cells of the J774 and Jurkat lines. HlyIICTD in a water solution is able to form oligomeric structures. In the presence of a membrane, HlyIICTD exits in oligomeric form, while monomeric forms are almost completely absent. HlyIICTD trimerizes in the presence of 4M urea, possibly forming some structure that can be integrated into the artificial bilayer membrane with the formation of pores. The current–voltage characteristic of these channels was determined. Such protein structures are characteristic of trimeric autotransporter proteins [3]. In this case, the secreted full-sized monomeric form of hemolysin II acts as a passenger, and HlyIICTD acts as an element involved in adhesion to the membrane and secretion from bacterial cells. The materials presented in this paper demonstrate that hemolysin II may belong to trimeric autotransporter proteins—the first case of the description of this family of molecules among Gram-positive microorganisms.

**Keywords:** hemolysin; autotransporter proteins; secretion; artificial bilayer membrane; pore forming

**Acknowledgments:** The study was supported by a grant from the Russian Foundation for Basic Research (no. 14-04-00592) and by the Ministry of Science and Higher Education of the Russian Federation (unique project no. RFMEFI60419X0218).

**References**

1. Baida G.; Budarina, Z.I.; Kuzmin, N.P.; Solonin, A.S. Complete nucleotide sequence and molecular characterization of hemolysin II gene from Bacillus cereus. *FEMS Microbiol.* **1999**, *180*, 7–14.
2. Rudenko, N.V.; Karatovskaya, A.P.; Zamyatina, A.V.; Siunov, A.V.; Andreeva-Kovalevskaya, Z.I.; Nagel, A.S.; Brovko, F.A.; Solonin, A.S. C-Terminal Domain of Bacillus cereus Hemolysin II Is Able to Interact with Erythrocytes. *Russ. J. Bioorg. Chem.* **2020**, *46*, 321–326, doi:10.1134/S1068162020030188.
3. Kiessling, A.R.; Malik, A.; Goldman, A. Recent advances in the understanding of trimeric autotransporter adhesins. *Med Microbiol. Immunol.* 2020, *209*, 233–242, doi:10.1007/s00430-019-00652-3.

### 9.2. Size Matters: An Evaluation, on a Molecular Basis, of Ontogenetic Modifications in the Composition of Bothrops jararacussu Snake Venom

Freitas-de-SousaLuciana^1^*NachtigallPedro^2^Portes-JuniorJosé Antônio^1^HoldingMatthew L^3^NystromGunnar S^3^EllsworthSchyler A.^3^GuimarãesNoranathan da Costa^1^TioyamaEmilly^1^OrtizFlora^4^SilvaBruno Rocha da^4^KunzTobias Saraiva^4^Junqueira-de-AzevedoInácio de Loiola Meirelles^5^GrazziotinFelipe Gobbi^6^RokytaDarin R^3^Moura-da-SilvaAna Maria^7^1Programa de Pós-Graduação em Ciências-Toxinologia, Laboratório de Imunopatologia, Instituto Butantan, São Paulo, Brazil2Laboratório Especial de Toxinologia Aplicada, Instituto Butantan, São Paulo, SP, Brazil3Department of Biological Science, Florida State University, Tallahassee, Florida 32306, USA4Laboratório de Coleções Zoológicas, Instituto Butantan, São Paulo, SP, Brazil5Laboratório Especial de Toxinologia Aplicada, Instituto Butantan, São Paulo, Brazil6Laboratório Especial de Coleções Zoológicas, Instituto Butantan, São Paulo, Brazil7Laboratório de Imunopatologia, Instituto Butantan, São Paulo, Brazil*Correspondence: luciana.sousa@butantan.gov.br

Ontogenetic changes in venom composition have been described in Bothrops snakes, but only a few studies have attempted to identify the targeted paralogues or the molecular mechanisms involved in venom modifications of gene expression during ontogeny. In this study, we decoded *B. jararacussu* venom gland transcripts from 6 specimens of varying sizes and analyzed the variability in the composition of independent venom proteomes from 19 individuals. We identified 125 distinct putative toxin transcripts, and of these, 73 were detected in venom proteomes and only 10 were involved in ontogenetic changes. Ontogenetic variability was linearly related to snake size and did not correspond to the maturation of the reproductive stage. Changes in the transcriptome were highly predictive of changes in the venom proteome. The basic myotoxic phospholipases A2 (PLA2s) were the most abundant components in larger snakes, while in venoms from smaller snakes, PIII-class SVMPs were the major components. The SVMPs identified corresponded to novel sequences and conferred both pro-coagulant and hemorrhagic functions to the venoms of small snakes. The mechanisms modulating venom variability are predominantly related to transcriptional events and may be related to the advantage of coagulant and hemorrhagic venoms of small snakes to predatory function.

**Keywords:***Bothrops jararacussu*; ontogenetic variability; transcriptome; proteome; 54 metalloproteinases; phospholipases A2

### 9.3. First Exploration of the Mesobuthus Cyprius Venom

HapeshiEvroula^1^*KyriacouSotirios^2^PanagiotidisMichail^2^PrimikyriAlexandra^3^GalatouEleftheria^1^NicolaidouVicky^1^ZachariaLefteris^1^EleftheriouConstantina^1^TsinoglouSocrates^4^SarigiannisYiannis^1^1Department of Life & Health Sciences, University of Nicosia, Nicosia, Cyprus2Department of Electron Microscopy/Molecular Pathology, The Cyprus Institute of Neurology and Genetics, Nicosia, Cyprus3Department of Chemistry, University of Ioannina, Ioannina, Greece4MedVenom Ltd., Nicosia, Cyprus*Correspondence: hapeshis.e@unic.ac.cy

The evolutionary history of scorpions begun around 425–450 million years ago, in the middle Silurian. More than 1500 species have been recognized and classified into different families. Mesobuthus cyprius, one of the two endemic scorpions in Cyprus, belongs to the Buthidae family, which is geographically distributed worldwide and is the largest of the scorpion families. Moreover, from a clinical perspective, Buthidae is the most important scorpion family as several members of this family are toxic to mammals and can be dangerous to humans. Even though Mesobuthus cyprius was discovered in 2000 using molecular phylogenetics, there are no other published data regarding the peptide and protein composition, toxicity, or any other activity of its venom. For this research work, several specimens were collected, and their venom composition was studied using liquid chromatography–tandem mass spectrometry (LC-PDA-MS and ultra-high-performance liquid chromatography (UPLC)-TOF-MS) techniques. Furthermore, a comparison of the venom of Mesobuthus cyprius with the venom of *Mesobuthus gibbosus*, the closest member of the family, common in Greece and Turkey, was performed. The same venoms were studied with solution-state nuclear magnetic resonance (NMR) spectroscopy. Finally, we tested the venom for its ability to cause cell death in a number of cancer cell lines.

**Keywords:** Mesobuthus cyprius; *Mesobuthus gibbosus*; scorpion venoms; scorpion toxins; UPLC-TOF-MS

## 10. Acknowledgments

The 1st International Electronic Conference on Toxins (IECT2021) was organized by the *Toxins* journal (https://www.mdpi.com/journal/toxins), which is published by MDPI.

The European Uremic Toxins (EUTox) Work Group, the French Society on Toxinology (SFET), the International Society for Mycotoxicology (ISM), and the Japanese Society of Mycotoxicology (JSMYCO) are also acknowledged for their support of this conference.

